# Improved Systemic Immunochemotherapy Employing an Oxaliplatin‐TLR7/8 Agonist Prodrug Strategy

**DOI:** 10.1002/advs.202514588

**Published:** 2026-04-13

**Authors:** Michael Gutmann, Martijn Dijkstra, Philipp Salomon, Jamie Cowles, Anja Federa, Dina Baier, Carola Jaunecker, Iemima Semerean, Venla Karvonen, Christine Pirker, Maria Sibilia, Dietmar Herndler‐Brandstetter, Petra Heffeter, Bernhard K. Keppler, Christian R. Kowol, Walter Berger

**Affiliations:** ^1^ Medical University of Vienna Center for Cancer Research Vienna Austria; ^2^ Comprehensive Cancer Center Vienna Medical University & General Hospital Vienna Austria; ^3^ Research Cluster “Translational Cancer Therapy Research” Vienna Austria; ^4^ University of Vienna Faculty of Chemistry, Institute of Inorganic Chemistry Vienna Austria; ^5^ University of Vienna Vienna Doctoral School in Chemistry (DoSChem) Vienna Austria

**Keywords:** albumin, gardiquimod, immunochemotherapy, maleimide, oxaliplatin, platinum(IV) prodrugs, TLR7/8, tumor‐targeted

## Abstract

Systemic application of Toll‐like receptor 7/8 (TLR7/8) agonists against cancer is severely limited due to uncontrolled immune activation. In this study, a platinum(IV)‐based prodrug strategy is developed for the systemic administration of a TLR7/8 agonist, selectively activated in the malignant tissue simultaneously with the immunogenic cell death inducer oxaliplatin. Two oxaliplatin(IV)‐based complexes are synthesized comprising the TLR7/8 agonist gardiquimod: Ox‐Gardi‐PEG, containing polyethylene glycol as the second axial ligand, and Ox‐Gardi‐Mal, a maleimide‐bearing derivative to exploit the tumor‐targeting effects of serum albumin. In vitro, cytotoxicity and immune pathway‐inducing potency of Ox‐Gardi‐PEG and Ox‐Gardi‐Mal are diminished under standard cell culture conditions compared to free oxaliplatin and gardiquimod, respectively, and markedly enhanced under reducing conditions, underscoring the activation‐by‐reduction concept. In vivo, Ox‐Gardi‐Mal shows superior and TLR7/8 signaling‐dependent anticancer efficacy and prolongs overall survival of cancer‐bearing mice, while mitigating hematotoxic effects associated with oxaliplatin. Therapy significantly elevates expression of MHC‐I on antigen‐presenting immune cell subsets, increases the frequency of activated plasmacytoid dendritic cells and tumor‐infiltrating CD8^+^ T cells, as well as depleted primarily immunosuppressive M2 macrophages. These results demonstrate that tumor‐targeted oxaliplatin(IV)‐based prodrugs carrying TLR7/8 agonists offer a potent dual‐release strategy for improved immunochemotherapy, while minimizing excessive immune responses associated with systemic TLR7/8 activation.

## Graphical abstract

A platinum(IV) prodrug is developed to systemically deliver the TLR7/8 agonist gardiquimod. Tumor‐targeting is mediated by an albumin‐binding maleimide, which limits premature complex activation. **Ox‐Gardi‐Mal** accumulates specifically in the tumor, where it activates the immune system. Therapy significantly prolongs overall survival in a colorectal cancer mouse model, while side effects of free TLR7/8 agonist and conventional oxaliplatin(II) therapy are avoided. Graphical scheme was created with BioRender.com

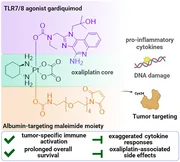

## 1 Introduction

Toll‐like receptor (TLR) agonists are immunomodulatory agents that have garnered increasing attention as potent anticancer therapeutics, both as monotherapies and in combination with cytotoxic chemotherapies [1, 2, 3, 4]. They target pattern recognition receptors of the innate immune system, which are essential for pathogen detection and the initiation of innate and, subsequently, adaptive immune responses [5]. Numerous TLR agonists have entered clinical trials for solid tumors [1, 2, 4, 6], however, only three are FDA‐approved: Bacillus Calmette‐Guérin (BCG) (TLR2, TLR4, partially TLR9), a bacterial preparation for the local treatment of bladder cancer [7], monophosphoryl lipid A (TLR4) as an adjuvant in human papillomavirus (HPV) vaccination [8], and imiquimod (R837) (TLR7), an imidazoquinoline derivative for topical treatment of skin diseases including actinic keratosis and superficial basal cell carcinoma [9]. TLR7 recognizes single‐stranded RNA in endosomes and activates, via the adapter protein primary‐response protein 88 (MyD88) and the transcription factor nuclear factor kappa‐light‐chain‐enhancer of activated B cells (NF‐κB), activator protein 1 (AP‐1) and interferon regulatory factors (IRFs), finally triggering the expression of inflammatory cytokines and interferons [10, 11]. TLR7 is primarily expressed in plasmacytoid DCs (pDCs) and B cells, and to a lesser extent in macrophages, monocytes, and NK cells. The closely related TLR8 is, with respect to myeloid cells, mainly found in monocytes, macrophages, and neutrophils, and less prominent in pDCs [12]. Based on the clinical success of imiquimod, several derivatives have been developed to improve antitumor efficacy, including resiquimod (R848) [13] and gardiquimod [14, 15]. While R848 has been intensively studied (pre)clinically [2, 3], gardiquimod remains comparably under‐investigated with limited preclinical data [16, 17]. Nevertheless, gardiquimod is a more potent derivative of imiquimod, selective for human and mouse TLR7 at < 1 µg mL^−1^, and only weakly active against human TLR8 (> 3 µg mL^−1^) [18].

Multiple reports suggested that TLR7/8 activators exert synergistic interactions in combination with platinum‐based chemotherapeutics in preclinical models of several cancer entities. Combination approaches of free platinum‐based chemotherapeutics included cisplatin combined with the TLR7/8 agonist CMA‐R848 in gastric cancer [19] or the TLR7/8/9 agonist CR108 ((S)‐2‐(2‐hydroxy‐3‐methylbutylthio)naphthalene‐1,4‐dione), a vitamin K3 derivative inducing reactive oxygen species (ROS) and mitochondrial dysfunction [20]. CR108, in combination with platinum‐based agents, increased the repertoire of tumor antigens [21] and induced formation of tertiary lymphoid structures (TLS) [22]. Furthermore, oxaliplatin in combination with imiquimod promoted antitumor immune responses against colorectal cancer [23]. Moreover, Verschuere et al. reported promising anticancer activity by combining amphiphilic conjugates of oxaliplatin and the imidazoquinoline IMDQ [24]. Concerning nanoformulations against colorectal cancer, liposomal oxaliplatin either directly contained IMDQ [25], or was combined with free R848 [26]. Additionally, oxaliplatin together with R848 reversed oxaliplatin resistance in colorectal cancer by repolarizing myeloid‐derived suppressor cells into anti‐tumorigenic M1 macrophages [27]. Among the worldwide approved platinum(II) drugs, oxaliplatin is of particular interest for combination with immunomodulatory drugs, like TLR agonists, as it is considered a bona fide inducer of immunogenic cell death (ICD) [28, 29]. ICD is characterized by the release of damage‐associated molecular patterns that can trigger adaptive and memory T cell responses, augmented for example by TLR7/8‐mediated innate immunity [23, 24, 25, 26, 27].

Given the strong immune‐activating potency associated with systemic administration of TLR7/8 agonists, novel application modalities are critical to ensure safe and tumor‐targeted activation strategies. While imiquimod has been successfully used as a topical treatment, systemic application is not tolerated based on the high risk of immune hyperactivation and inflammation [30]. A potential strategy to circumvent these limitations involves the use of platinum(IV) prodrugs. These experimental chemotherapeutic compounds are comparably substitution inert, attenuating their reactivity and reducing dose‐limiting toxicities associated with platinum(II) therapy [31, 32]. They also represent promising drug candidates to address platinum drug resistance [33]. The preclinically most advanced platinum(IV) compound is satraplatin which, however, failed in a clinical phase III study due to rapid reduction in red blood cells [34]. This highlights the necessity for improved and tumor‐targeted platinum(IV) prodrug designs. The versatile prodrug system additionally allows the incorporation of bioactive axial ligands, enabling co‐delivery of chemotherapeutics and highly active immunomodulatory agents [35, 36]. Recently, first examples of integrating TLR7/8 agonists as axial ligands in platinum(IV) scaffolds have been reported to enable systemic delivery. For example, oxaliplatin(IV)‐based prodrugs incorporating the TLR7 agonists SZU101 [37] demonstrated promising preclinical anticancer activity. However, these prodrug strategies lack tumor‐specific targeting and may lead to exacerbated TLR7/8 activation and adverse immune responses.

Controlled‐release drug delivery systems represent an approach to address these limitations [38], as demonstrated by cisplatin(IV) prodrugs containing IMDQ, which is sequentially released via a glutathione‐ and a γ‐glutamyltranspeptidase (GGT)‐responsive mechanism. However, this concept depends on elevated GGT expression, limited to certain cancer types [39]. To expand the applicability of the delivery system across a broader range of cancer types, passive tumor‐targeting strategies have been developed, including conjugation of platinum(IV) prodrugs to serum albumin via maleimide moieties. This approach prolongs the plasma half‐life and enhances tumor accumulation of the prodrug by exploiting the increased albumin consumption, as well as the enhanced permeability and retention (EPR) effect of the tumor tissue [36, 40, 41, 42, 43, 44]. The success of Abraxane, an albumin‐bound paclitaxel formulation [45] approved for breast [46] and lung cancer [47], exemplifies this approach.

In this study, we designed an oxaliplatin(IV)‐based prodrug functionalized for albumin‐targeting and tumor‐specific release of the TLR7/8 agonist gardiquimod, focusing on colorectal cancer due to the central role of oxaliplatin in treatment of this devastating disease. Cell‐free and in vitro analyses proved suitable stability, reduction kinetics, and albumin‐binding properties and confirmed the prodrug characteristics of the developed platinum(IV) complexes. Preferential pharmacokinetic characteristics allowed the systemic application of gardiquimod doses at least 7.4‐fold above the maximal tolerated dose of the free drug, without inducing signs of systemic immune hyperactivation in a syngeneic colorectal cancer mouse model. Oxaliplatin‐mediated hematotoxicity was distinctly reduced by this prodrug approach. While CD8^+^ T cell infiltration and immune cell activation in the tumor tissue were significantly induced, primarily tumor‐promoting M2 macrophages were depleted. Finally, maleimide‐mediated albumin‐targeting markedly enhanced tumor accumulation, as well as anticancer activity, and prolonged overall survival of the treated animals in a TLR7/8‐dependent manner.

## 2 Results and Discussion

### 2.1 Chemical Synthesis, Reduction, and Albumin Binding

**SCHEME 1:**
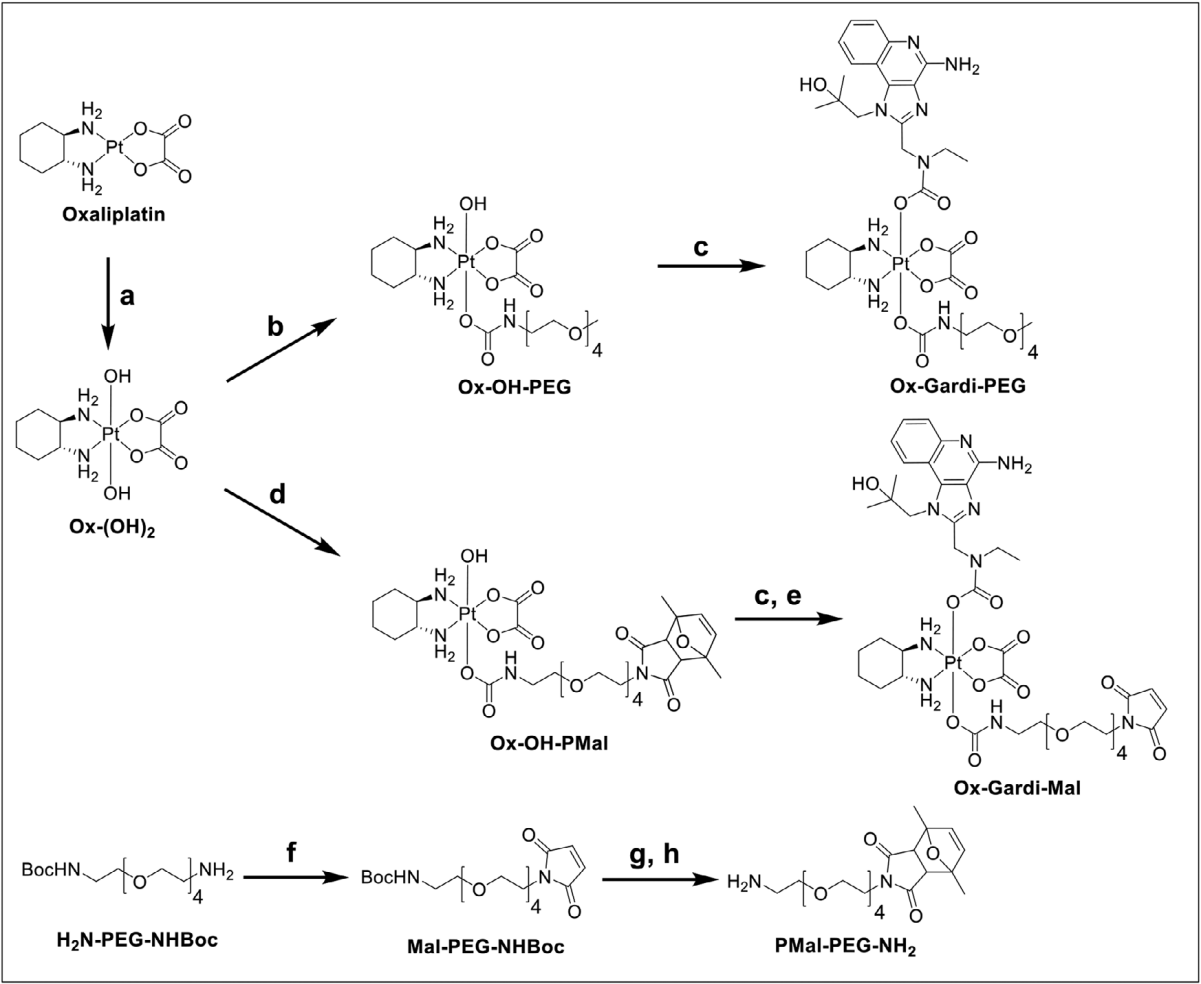
Synthetic routes for the oxaliplatin(IV)‐gardiquimod complexes: (a) H_2_O_2_ (50 wt.%), MQ‐H_2_O, room temperature (RT); (b) 3,6,9,12‐tetraoxatridecanamine, DSC, DMSO, RT; (c) Gardiquimod (Gardi), DSC, DMF, RT; (d) PMal‐PEG‐NH_2_, DSC, DMSO, RT; (e) DMSO, 90°C; (f) methyl 2,5‐dioxo‐2,5‐dihydro‐1*H*‐pyrrole‐1‐carboxylate, saturated NaHCO_3_ solution, 0°C – RT; (g) 2,5‐dimethylfurane, acetonitrile (ACN), 60°C; (h) 1.25 M HCl, EtOH, RT.

Oxaliplatin was synthesized [48] and oxidized to Ox‐(OH)_2_ with H_2_O_2_ via well‐known literature procedures [49]. To ensure sufficient aqueous solubility of the gardiquimod‐functionalized oxaliplatin(IV) complex, a polyethylene glycol (PEG) linker was attached to the second axial position. Therefore, 3,6,9,12‐tetraoxatridecanamine was axially coupled via a carbamate linkage by using the peptide coupling agent *N,N*‐disuccinimidyl carbonate (DSC) to obtain the precursor complex Ox‐OH‐PEG (Scheme 1).

Gardiquimod was conjugated via its secondary amine functionality to Ox‐OH‐PEG with a similar DSC‐mediated carbamate linkage to synthesize the final complex Ox‐Gardi‐PEG. To develop a maleimide‐functionalized gardiquimod‐containing oxaliplatin(IV) derivative for endogenous albumin binding, a recently reported synthetic strategy utilizing a protected maleimide was exploited. In more detail, *tert*‐butyl (14‐amino‐3,6,9,12‐tetraoxatetradecyl)carbamate (H_2_N‐PEG‐NHBoc) was coupled with a maleimide functionality using methyl 2,5‐dioxo‐2,5‐dihydro‐1*H*‐pyrrole‐1‐carboxylate in saturated sodium bicarbonate solution, and subsequently the maleimide was protected via a Diels‐Alder reaction with 2,5‐dimethylfurane. The Boc‐group was deprotected with 1 M HCl in EtOH and neutralized with a slight excess of triethylamine to yield PMal‐PEG‐NH_2_ (Scheme 1). Subsequently, first PMal‐PEG‐NH_2_ and afterwards gardiquimod were coupled to Ox‐(OH)_2_, both times using DSC. Finally, the maleimide moiety was deprotected by release of 2,5‐dimethylfurane via a retro‐Diels alder reaction at 90°C, which afforded Ox‐Gardi‐Mal after preparative high‐performance liquid chromatography (HPLC) purification. Both complexes were analyzed in detail by 1D and 2D NMR spectrometry, mass spectrometry, and the purity was confirmed by HPLC measurements and elemental analysis (see Experimental Section and Figures S1–S3). The long‐term stability of the solid Ox‐Gardi‐PEG and Ox‐Gardi‐Mal compounds was investigated at different time points (0, 24 h, 1 and 4 weeks) and temperatures (–20°C, 4°C, and 40°C) and analyzed by HPLC measurements. The data reveal high stability of both complexes, with no visible changes over 4 weeks at –20°C and 4°C, and >98% intact complex even after 4 weeks at 40°C (Figure S4).

**FIGURE 1:**
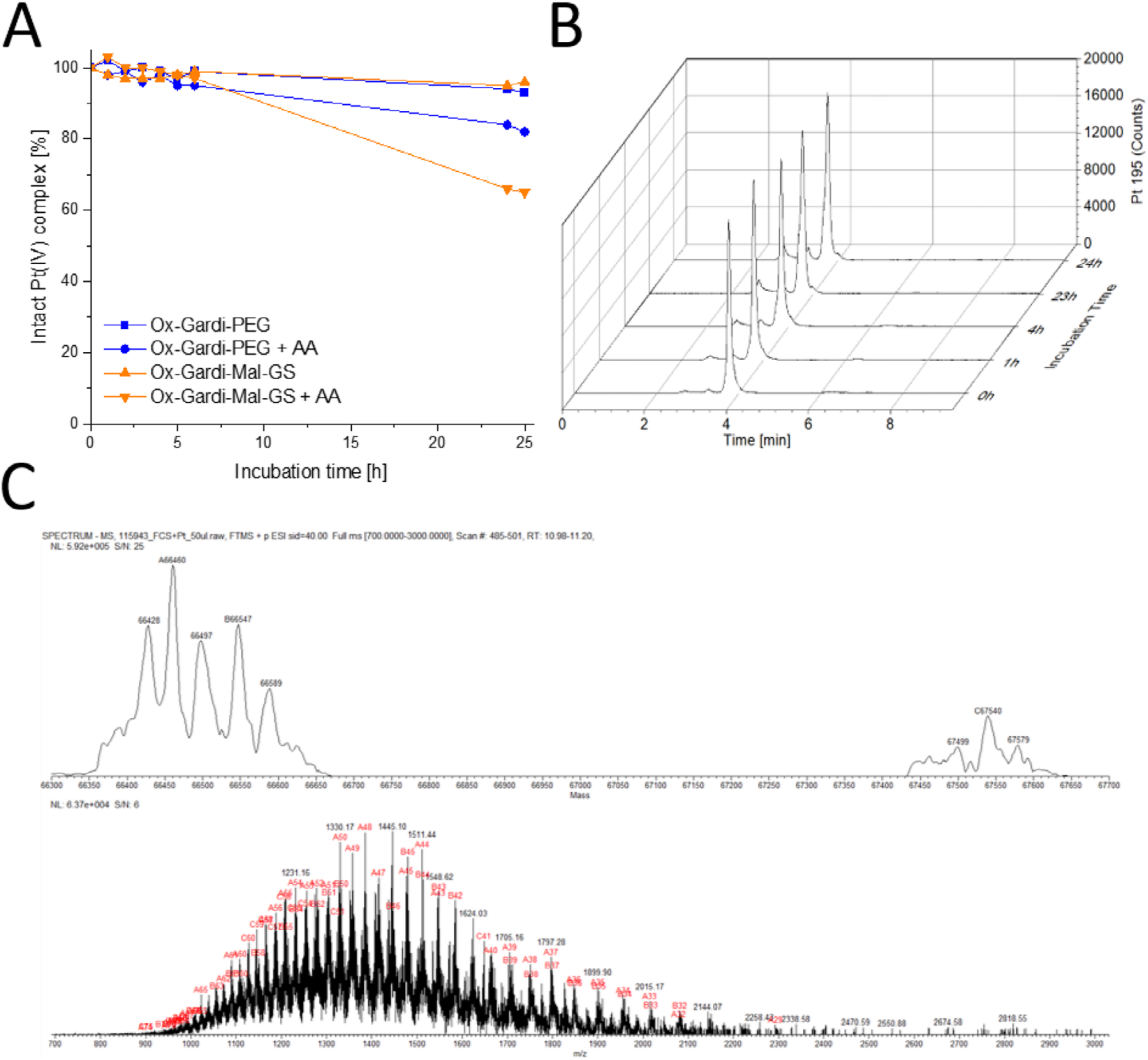
Stability and reduction kinetics of Ox‐Gardi‐PEG, Ox‐Gardi‐Mal‐GS and albumin‐binding properties of Ox‐Gardi‐Mal. (A) Stability and reductive properties of 1 mM Ox‐Gardi‐PEG in 150 mM PB (pH 7.4) at 20°C over 25 h with or without 10 eq. AA, measured with UHPLC. 1 mM Ox‐Gardi‐Mal was first incubated with 1 eq. GSH in 150 mM PB (pH 7.4) to avoid maleimide hydrolysis and subsequently incubated with ± 10. eq. AA at 20°C over 25 h. (B) ^195^Pt‐traces of 50 µM Ox‐Gardi‐Mal in fetal calf serum (FCS) containing 150 mM PB, pH 7.4 at 37°C over 24 h, measured with SEC‐ICP‐MS. (C) HPLC‐ESI‐MS spectrum of the albumin‐containing serum fraction incubated with 50 µM Ox‐Gardi‐Mal for 2 h (drug:albumin ratio ∼1:12) and the respective deconvoluted peaks (native albumin at 66428 Da; Ox‐Gardi‐Mal‐albumin at 67540 Da). *AA = L‐ascorbic acid; Ox‐Gardi‐Mal‐GS = glutathione adduct of Ox‐Gardi‐Mal*.

First the stability and reductive properties of Ox‐Gardi‐PEG were investigated by incubation in phosphate buffer (PB) at pH 7.4 at 20°C over 25 h in the absence and presence of 10 eq. of reducing agent L‐ascorbic acid (AA) as model reductant. The amount of parental complex was monitored with ultra‐high performance liquid chromatography (UHPLC) (Figure 1A). Following a 24 h incubation period, Ox‐Gardi‐PEG was stable with >95% left in the absence of a reducing agent, compared to 80% in the presence of AA. The respective chromatogram (Figure S5A) also clearly indicated the release of gardiquimod and oxaliplatin, although the latter is hard to detect due to the very low absorption coefficient. These results are well in accordance with other oxaliplatin(IV) prodrugs showing slow reduction in the presence of AA [42, 44]. Ox‐Gardi‐Mal could not be directly included in this assay due to the well‐known hydrolysis of maleimides at physiological pH (Figure S5B) [50]. Therefore, Cys34 albumin binding was simulated by incubation with one eq. of glutathione (GSH) at pH 7.4, leading to the immediate formation of a new peak corresponding to Ox‐Gardi‐Mal‐GS (Figure S5C). Subsequently, again 10 eq. AA was added and the reduction monitored at 20°C over 25 h. The overall kinetics were comparable to Ox‐Gardi‐PEG, although a slightly higher reduction was observed after 25 h (∼65%). For comparison, platinum(IV) complexes with a cisplatin core are reduced much faster, typically being completely reduced within ∼10 h [40].

We confirmed the activation by reduction of the platinum(IV) complexes also in the presence of GSH in cell‐free conditions (Figure S6A). Additionally, the prodrugs were spiked into cell or tumor lysates and incubated for 48 h at 37°C and gardiquimod was analyzed by HPLC‐MS. Especially in tumor lysates, a pronounced release of gardiquimod from both platinum(IV) complexes was observed reaching ∼ 80% after 48 h (Figure S6B). These data suggest that cellular or extracellular components of the tumor microenvironment (TME) might directly contribute to the reductive release of gardiquimod in the malignant tissue.

The albumin‐binding properties of Ox‐Gardi‐Mal (Figure 1B) were studied by incubation in phosphate‐buffered fetal calf serum (FCS) at 37°C over 24 h and compared to Ox‐Gardi‐PEG (Figure S7A). ^195^Pt traces were measured via size exclusion inductively coupled plasma mass spectrometry (SEC‐ICP‐MS). Serum proteins have a retention span of ∼2–4 min as part of the high molecular weight fraction, with albumin at ∼4.0 min (Figure S7B). After incubation with Ox‐Gardi‐Mal, already at the 0 h time point ∼99% of the platinum was bound to albumin, and the albumin‐drug conjugate was stable over 24 h without release of platinum from the protein (Figure 1B). The remaining 1–2% in the low‐molecular‐weight fraction (LMWF > 4 min) are likely traces of hydrolyzed maleimide complexes unable to bind to albumin. In contrast, the reference complex Ox‐Gardi‐PEG, lacking the ability to conjugate to albumin, was found mainly in the LMWF, even after extended incubation time up to 24 h (Figure S7A). To prove the covalent binding of Ox‐Gardi‐Mal to albumin, the respective serum fraction was collected and analyzed by HPLC‐ESI‐MS measurements (drug:albumin ratio ∼1:12). The deconvoluted spectrum clearly indicated beside the native albumin at 66428 Da a second peak at 67540 Da, correlating with the mass of albumin in conjugation with Ox‐Gardi‐Mal (Figure 1C; for the pure albumin reference see Figure S7C).

### 2.2 Cytotoxic Activity

**FIGURE 2:**
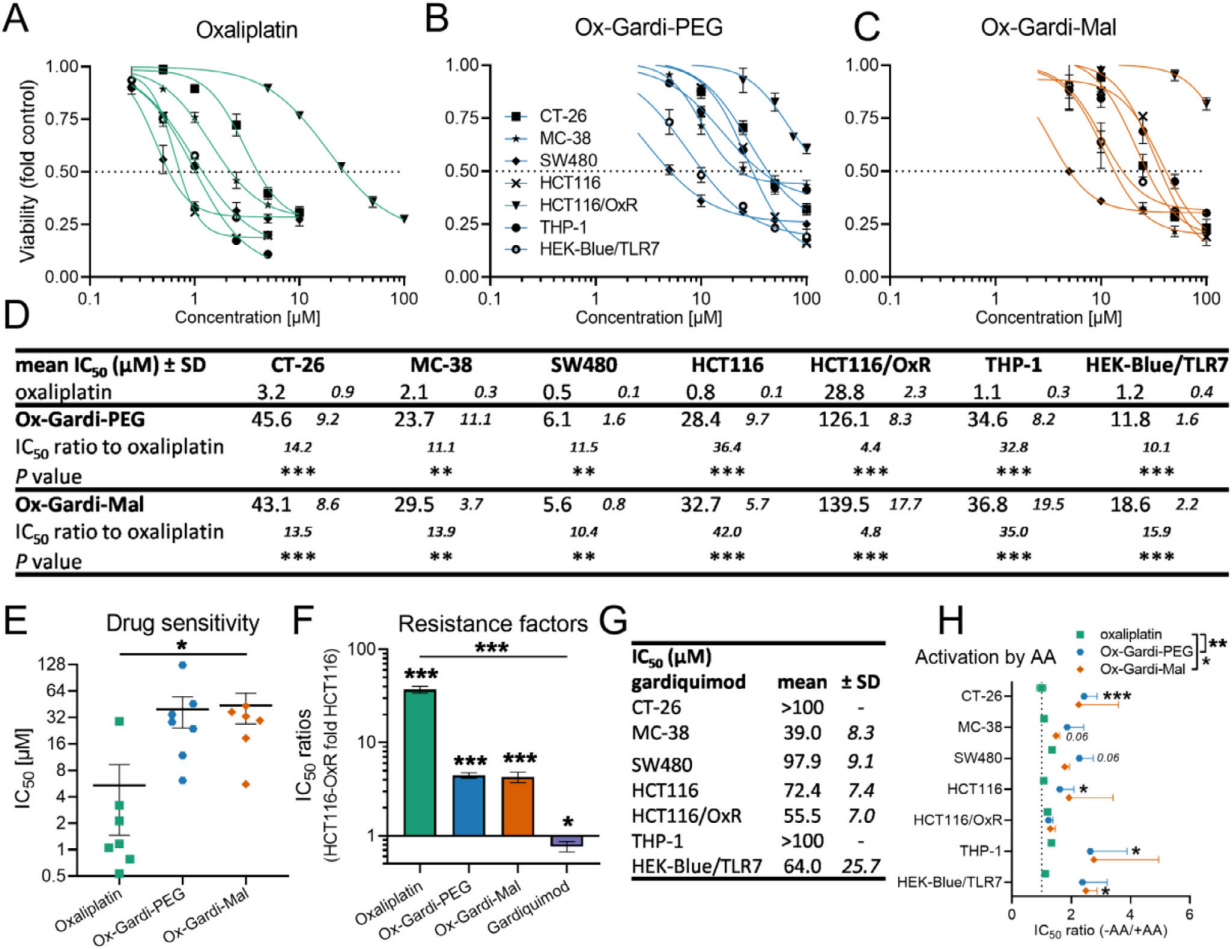
Cytotoxic activity of Ox‐Gardi‐PEG and Ox‐Gardi‐Mal in cancer cell models compared to oxaliplatin/gardiquimod and impact of reducing conditions. (A‐H) Impact of 72 h drug treatment on the viability of the indicated cancer cell lines, determined by MTT‐based cell viability assay. (A–C) Dose‐response curves of oxaliplatin (A), Ox‐Gardi‐PEG (B), and Ox‐Gardi‐Mal (C) modelled using the four‐parameter logistic (4PL) non‐linear regression model. One representative experiment out of at least three performed is shown. (D) Table of IC_50_ values, derived from dose‐response‐curves by interpolation, and IC_50_ ratios. (E) IC_50_ values are depicted for all investigated cell models. (F) Resistance factors (IC_50_ ratio) were calculated for each compound between the parental HCT116 and the oxaliplatin‐resistant subline HCT116/OxR. (G) IC_50_ table showing the impact of gardiquimod on the viability of the indicated cancer cell lines. (H) Impact of reducing conditions (AA; 50 µM) on the cytotoxicity of oxaliplatin, Ox‐Gardi‐PEG, and Ox‐Gardi‐Mal shown as IC_50_ ratio between –AA and +AA. Data points are depicted as mean ± SD. The statistical significance of differences in IC_50_ ratios (D) and IC_50_ values between Ox‐Gardi‐PEG, Ox‐Gardi‐Mal, and oxaliplatin (E), the resistance factors between the indicated drugs (F), and activation by AA across all cell models (H) was tested using one‐way ANOVA with the Tukey multiple comparison. The statistical significance of differences in IC_50_ ratios between HCT116 and HCT116/OxR cells (F) and between –AA and +AA (H) for the indicated cell models was tested using Student's *t*‐test. *IC_50_ = half‐maximal inhibitory concentration; AA = L‐ascorbic acid*. *P < 0.05; **P < 0.01; and ***P < 0.001.

**FIGURE 3:**
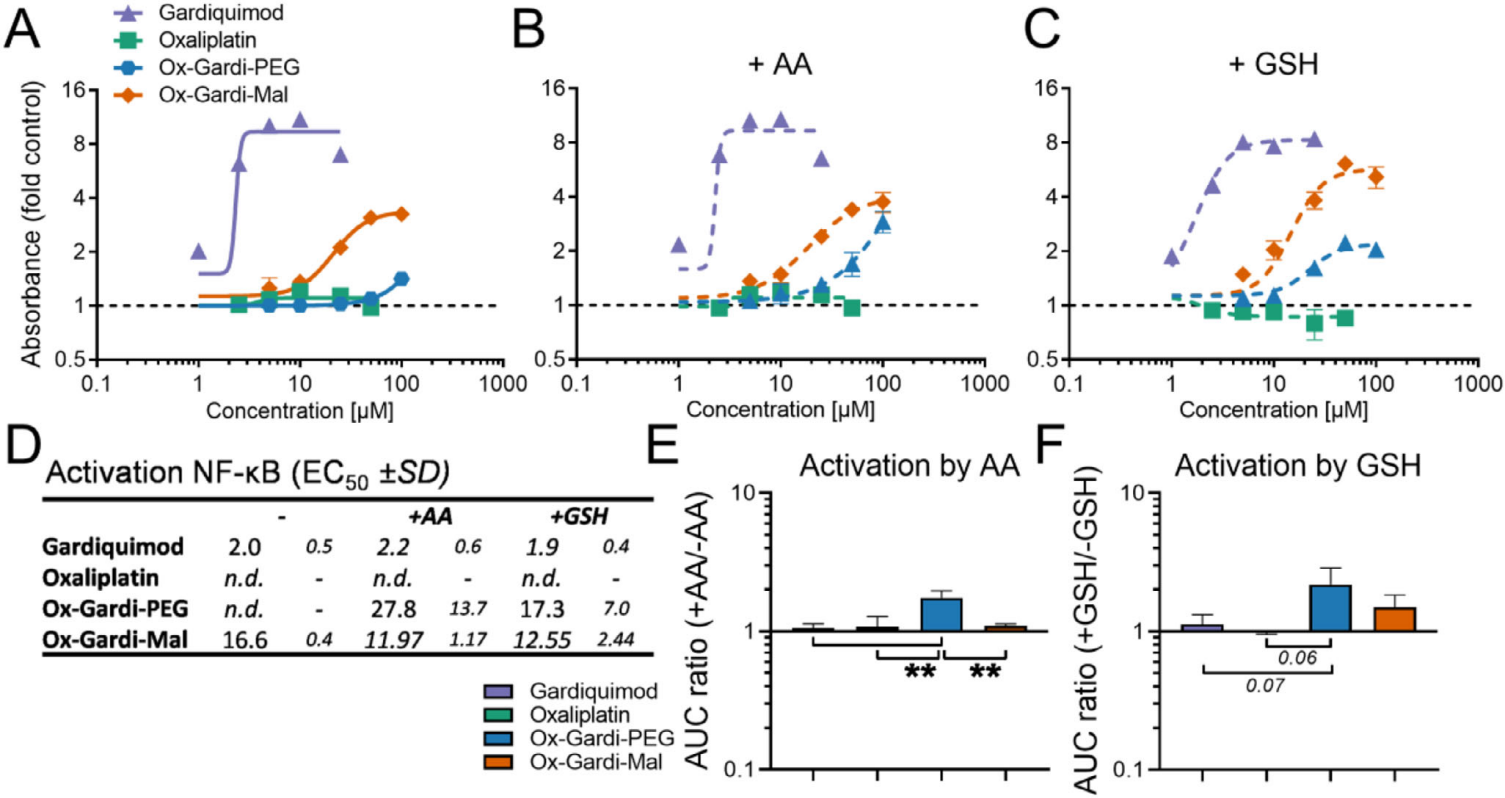
Gardiquimod‐mediated pathway induction by Ox‐Gardi‐PEG and Ox‐Gardi‐Mal compared to gardiquimod/oxaliplatin and impact of reducing conditions. (A–F) NF‐κB/AP‐1‐inducible secreted embryonic alkaline phosphatase (SEAP) reporter levels were determined by measuring the absorbance at 620 nm in the HEK‐Blue/hTLR7 cell culture supernatant following 18 h of treatment with the indicated drugs. (A–C) Dose‐response curves of the indicated drugs, modelled using the four‐parameter logistic (4PL) non‐linear regression model, under standard (A) and reducing (50 µM AA, in B and E) (10 mM GSH, in C and F) cell culture conditions. One representative experiment out of at least three performed is shown. (D) EC_50_ values of the investigated drugs under standard and reducing cell culture conditions. (B‐F) Impact of reducing conditions was calculated as the area under curve (AUC) ratio between + AA and – AA and between + GSH and – GSH. Data points are depicted as mean ± SD. The statistical significance of differences in the AUC ratio between + AA and – AA (E), and between + GSH and – GSH (F) was tested using one‐way ANOVA with the Tukey multiple comparison test. *AA = L‐ascorbic acid; GSH = glutathione; AUC = area under the dose‐response curve; EC_50_ = half‐maximal effective concentration;* **P < 0.01.

Previous studies have shown that activation‐dependent platinum(IV) prodrugs, which are kinetically highly stable against reduction, exerted distinctly lower cytotoxicity against cancer cells as compared to oxaliplatin in vitro, but subsequently achieved superior anticancer efficacy in the in vivo situation [41, 42, 43]. To evaluate whether the stability and reduction properties of Ox‐Gardi‐PEG and Ox‐Gardi‐Mal observed under cell‐free conditions (compare Figure 1A) corresponded to decreased cytotoxic activity against cancer cells, mean half‐maximal inhibitory concentration (IC_50_) values of the investigated drugs were derived from MTT‐based viability assays in several cell models (Figure 2). Notably, the precise in vitro evaluation of albumin‐mediated effects of Ox‐Gardi‐Mal is hampered by the low albumin concentration (∼33.8 µM) in cell culture medium, the pronounced reactivity of maleimides toward many thiol‐containing components, and the potential hydrolysis of maleimides [51, 52]. Nevertheless, both platinum(IV) prodrugs exerted widely comparable cytotoxic activities in the tested cell models (Figure 2D,E). Colorectal cancer cell lines of murine (CT‐26, MC‐38) and human (HCT116, HCT116/OxR, SW480) origin, human monocytic THP‐1 cells, as well as the HEK‐Blue/hTLR7 reporter cell model demonstrated sensitivity to oxaliplatin at low µM doses (Figure 2A,E), with IC_50_ values ranging from 0.8 to 28.8 µm (Figure 2D). Indeed, the cytotoxicity of Ox‐Gardi‐PEG (Figure 2B) and Ox‐Gardi‐Mal (Figure 2C) was in all tested cell models significantly lower compared to oxaliplatin (Figure 2D,E), with the activity reduced by 4.4‐ and 4.8‐fold (HCT116/OxR) to 36.4‐ and 42‐fold (HCT116) for Ox‐Gardi‐PEG and Ox‐Gardi‐Mal, respectively, at the IC_50_ level (Figure 2D). On average, the cell models exhibited a 17.2‐fold and a 19.4‐fold lowered sensitivity to Ox‐Gardi‐PEG and Ox‐Gardi‐Mal, respectively, as compared to oxaliplatin (Figure 2D). The HCT116/OxR subline, selected for acquired oxaliplatin resistance [53], was the least responsive cell line to oxaliplatin. In comparison with the parental HCT116 cells (IC_50_: 0.8 µM), a 37‐fold decreased vulnerability to oxaliplatin confirmed the resistance phenotype. In contrast, the activity reduction as compared to free oxaliplatin was only 4.4‐fold and 4.3‐fold for Ox‐Gardi‐PEG and Ox‐Gardi‐Mal, respectively, in the HCT116/OxR background of acquired oxaliplatin resistance (Figure 2D,F). This suggests that the oxaliplatin(IV) prodrugs are substantially less affected by established oxaliplatin resistance mechanisms. A cytotoxic/cytostatic activity of free gardiquimod was found within an IC_50_ range of 39 to >100 µM (Figure 2G). Consequently, viability‐reducing concentrations of the TLR7/8 agonist were observed only at doses several‐fold higher than the mean effective concentrations (EC_50_) required for NF‐κB pathway activation (compare Figure 3). Notably, the highest cytotoxicity of the TLR7/8 agonist was detected in MC‐38 cells (IC_50_: 39.0 µM) (Figure 2G). The higher cytotoxicity of gardiquimod in the oxaliplatin‐resistant HCT116/OxR subline compared to the parental cells (Figure 2F,G) suggests increased vulnerability to TLR7/8 agonists in the context of acquired oxaliplatin resistance. Co‐application of gardiquimod and oxaliplatin revealed no substantial synergistic interactions in the investigated cell models, apart from THP‐1 cells, which showed a weak synergism, probably reflecting the immune cell background (Figure S8).

Reduction of platinum(IV) complexes has been reported for small molecule reductants including AA and reduced glutathione (GSH), while in cell extracts the strongest reductive power was ascribed to high‐molecular weight protein fraction [19, 54]. To test reduction‐mediated prodrug activation, reducing conditions were simulated by co‐incubation with the mild reducing agent AA, GSH, and cell extracts (Figure 2H; Figure S9). While AA supplementation was not modulating the sensitivity of all investigated cell models toward oxaliplatin (Figure 2H; Figure S9A) and gardiquimod (Figure S9D–F), cytotoxicity was significantly enhanced in CT‐26, HCT116, and THP‐1 cells in case of Ox‐Gardi‐PEG, and in HEK‐Blue/TLR7 cells in case of Ox‐Gardi‐Mal (Figure 2H; Figure S9B,C). Co‐incubation of Ox‐Gardi‐PEG, Ox‐Gardi‐Mal, and oxaliplatin with GSH abrogated their cytotoxic activity in the CT‐26 cell model, likely due to extracellular GSH‐mediated inactivation or export of the GSH‐platinum(II) conjugate and/or attenuation of drug‐induced ROS (Figure S9G) [55, 56]. Supplementation of cell extracts, prepared from A2780 cells, significantly elevated cytotoxicity of Ox‐Gardi‐Mal, but not Ox‐Gardi‐PEG and oxaliplatin in CT‐26 cells (Figure S9H). In MC‐38 cells, AA co‐administration dampened the activity of gardiquimod (Figure S9F), while no effects were observed in the other cell models. In this case the reducing agent likely mitigated gardiquimod‐induced oxidative stress by scavenging ROS [57]. Cytotoxicity of gardiquimod remained comparably unaffected by GSH and cell extract supplementation in CT‐26 cells (Figure S9I).

To conclude, the markedly decreased in vitro anticancer activity of Ox‐Gardi‐PEG and Ox‐Gardi‐Mal compared to oxaliplatin was consistent with their high stability and slow reduction kinetics observed under cell‐free settings (compare Figure 1A), and confirmed their distinct stability also under standard cell culture conditions. The enhanced cytotoxic activity of both complexes upon co‐exposure with reducing agents supports the reduction‐triggered activation mechanism and delineates, in agreement with other reports [41, 42, 43], the prodrug concept.

### 2.3 Intracellular Platinum Accumulation

To evaluate differences in the uptake and export of the prodrugs versus oxaliplatin, the intracellular platinum content following treatment with Ox‐Gardi‐PEG, Ox‐Gardi‐Mal, and oxaliplatin was investigated in a panel of cancer cell lines by ICP‐MS (Figure S10). Cells were exposed for 3 h to assess the uptake of the intact platinum(IV) complexes, while minimizing cytotoxic effects. In all tested cell models, a dose‐dependent accumulation of both prodrugs and oxaliplatin was observed (Figure S10A). Treatment with the prodrugs resulted in significantly lower intracellular platinum levels compared to oxaliplatin, with most pronounced differences found in the case of CT‐26 and HCT116 cells (Figure S10B). Notably, while intracellular platinum levels were comparable between both prodrugs in HCT116, HCT116/OxR, and THP‐1 cells, Ox‐Gardi‐Mal was accumulating to a significantly higher extent than Ox‐Gardi‐PEG in CT‐26 cells, which can be explained by the generally high cellular albumin uptake reported for this cell model [58]. In the oxaliplatin‐resistant HCT116/OxR cell model, treatment with oxaliplatin led to significantly lower intracellular platinum levels than in the parental cells (Figure S10C), confirming a resistance phenotype associated with reduced drug accumulation [59]. When comparing platinum levels derived from oxaliplatin, Ox‐Gardi‐PEG, and Ox‐Gardi‐Mal at equimolar concentrations between resistant and parental HCT116 cells, the prodrugs were markedly less affected by the existing resistance mechanisms, resulting in similar or even higher platinum levels in the oxaliplatin‐resistant compared to the parental cells (Figure S10C). The significantly lowered prodrug‐derived platinum content points toward an altered cellular uptake mechanism of intact platinum(IV) complexes versus free oxaliplatin, and further supports the increased vulnerability of an oxaliplatin‐resistant background to oxaliplatin(IV) prodrugs.

### 2.4 Gardiquimod‐Mediated TLR7 Pathway Activation

TLR7/8 activation at endosomal membranes initiates a signaling cascade that activates NF‐κB, AP‐1, or IRF transcription factors, culminating in the production of inflammatory mediators, which determine the diverse cellular outcomes of TLR7/8 signaling. The TLR7 pathway‐inducing capacity of Ox‐Gardi‐PEG and Ox‐Gardi‐Mal was assessed in comparison to oxaliplatin and gardiquimod in a reporter cell model, engineered to co‐express human TLR7 and an NF‐κB/AP‐1‐inducible reporter (HEK‐Blue^TM^/hTLR7) (Figure 3). Gardiquimod potently triggered the NF‐κB/AP‐1 pathway, with EC_50_ values of approximately 2.0 (± 0.5) µM (Figure 3A,D), which corroborates other reports [60]. In contrast to the steep sigmoidal dose‐response curve induced by gardiquimod, Ox‐Gardi‐PEG was not able to significantly stimulate reporter production along the investigated concentration range under standard cell culture conditions (Figure 3A,D). Ox‐Gardi‐Mal moderately activated the NF‐κB pathway in the absence of reducing conditions, with EC_50_ values of 16.6 (± 0.4) µM (Figure 3A,D). These differences were not mediated by distinct cytotoxic effects of Ox‐Gardi‐PEG (Figure S11D). Treatment with oxaliplatin as single agent did not trigger pathway activation in this cell model (Figure 3A–C). Reducing conditions, obtained by supplementing AA or GSH, did not impact the pathway‐inducing ability of gardiquimod or oxaliplatin (Figure 3B,D,E). In case of Ox‐Gardi‐PEG, co‐exposure with AA markedly triggered reporter induction (Figure 3B), resulting in a significantly increased area under curve (AUC) ratio, which was not observed for the Ox‐Gardi‐Mal complex (Figure 3E). Co‐incubation of GSH distinctly elevated reporter induction by both prodrugs, which indicates activation of both platinum(IV) complexes by this reducing agent (Figure 3C,D,F). Application of the TLR7/8 receptor inhibitor enpatoran completely abrogated gardiquimod‐, as well as prodrug‐induced NF‐κB/AP‐1 pathway activation (Figure S11A–C). This effect was not based on inhibition of HEK‐Blue^TM^/hTLR7 cell viability as obvious from an enpatoran dose‐response curve (Figure S11E). Also, both platinum(IV) prodrugs did not reach IC_50_ values even when combined with AA as reductant within the short exposure time of 18 h (Figure S11D). Moreover, the addition of enpatoran to Ox‐Gardi‐Mal and AA did not further reduce cell viability (figure S11F; viability data for the experiment shown in Figure S11B). These data illustrate, that the massive inhibition of NF‐κB/AP‐1 pathway activation by enpatoran cannot be attributed to a cytotoxic effect but to actual TLR7/8 inhibition.

### 2.5 Anticancer Efficacy In Vivo

**FIGURE 4:**
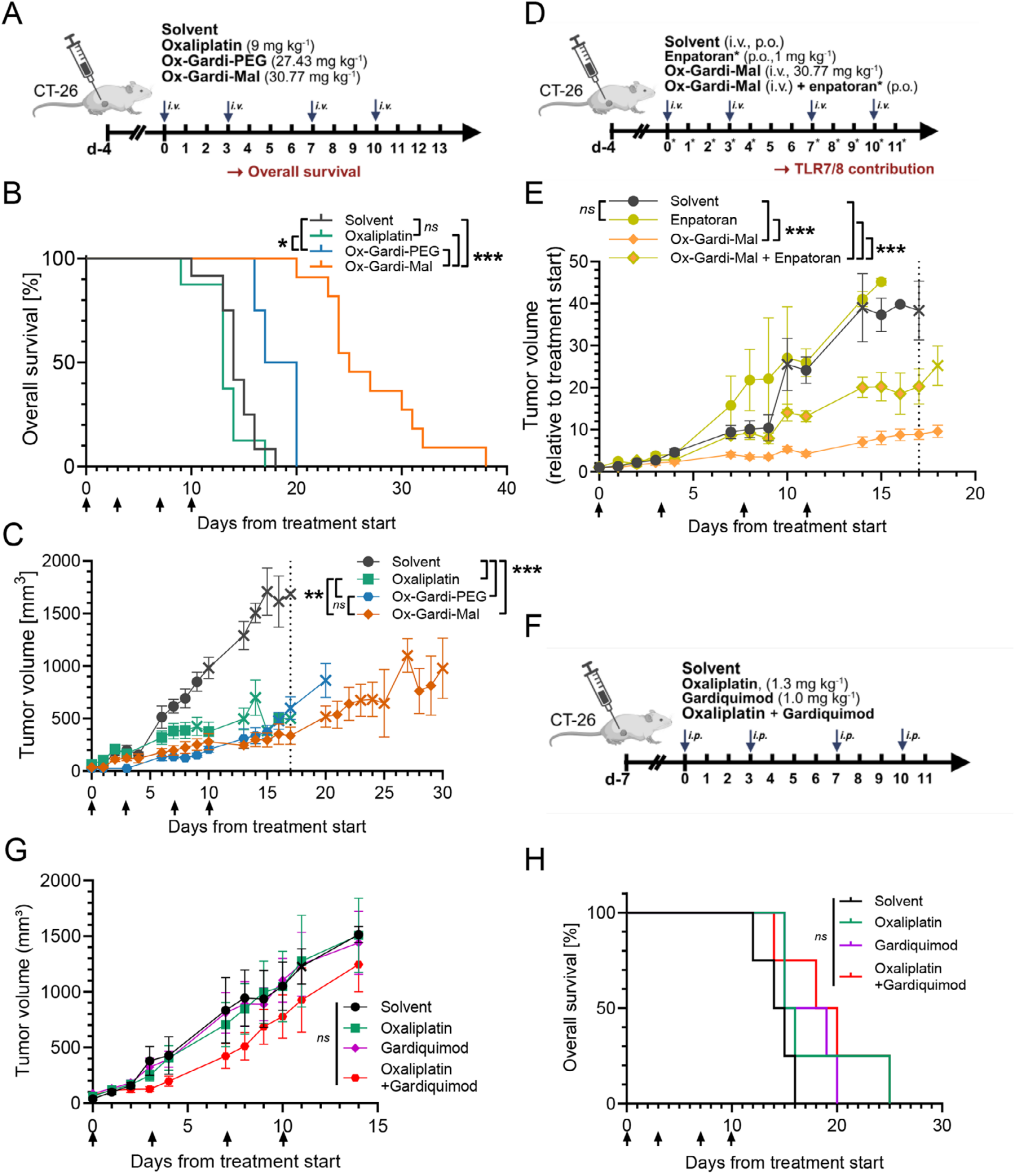
Anticancer efficacy of Ox‐Gardi‐PEG and Ox‐Gardi‐Mal compared to oxaliplatin without and with gardiquimod and impact of TLR7/8 inhibition in a syngeneic tumor mouse model. (A–C) CT‐26 colorectal cancer‐bearing BALB/c mice were dosed intravenously (i.v.) twice weekly for 2 weeks with Ox‐Gardi‐Mal (*n* = 12), Ox‐Gardi‐PEG (*n* = 4), or oxaliplatin (*n* = 8), equimolar to 9 mg kg^−1^ oxaliplatin. (D, E) CT‐26 colorectal cancer‐bearing BALB/c mice were dosed intravenously (i.v.) twice weekly for 2 weeks with Ox‐Gardi‐Mal (*n* = 4) equimolar to 9 mg kg^−1^ oxaliplatin as compared to solvent (*n *= 4). Mice were treated daily for 10 days with the TLR7/8 inhibitor enpatoran or solvent. (F–H) CT‐26‐bearing BALB/c mice were dosed intraperitoneally (i.p.) twice weekly for 2 weeks with premixed gardiquimod at the MTD of 1 mg kg^−1^ and the equimolar dose of 1.3 mg kg^−1^ of free oxaliplatin. (C,E,G) Tumor volumes, assessed via caliper measurements of mice treated with the indicated compounds, are depicted as mean ± SEM. Euthanasia of individual mice is indicated by an X symbol in place of a data point symbol. (B,H) Overall survival of mice is depicted in Kaplan‐Meier curves. Arrows below the x‐axis (B,C,E,G,H) indicate the days of drug application. The statistical significance of differences in overall survival was tested using a log‐rank (Mantel‐Cox) test. The statistical significance of differences in tumor volumes (in C and E until day 17 as indicated by the dashed line, in G until day 14) between the treatment groups was tested using two‐way ANOVA with the Sidak multiple comparison test. *i.v. = intravenous; p.o. = per os; i.p. = intraperitoneal; ns = non‐significant; SEM = standard error of mean*. *P < 0.05; **P < 0.01; and ***P < 0.001. Experimental schemes were created with BioRender.com.

**FIGURE 5:**
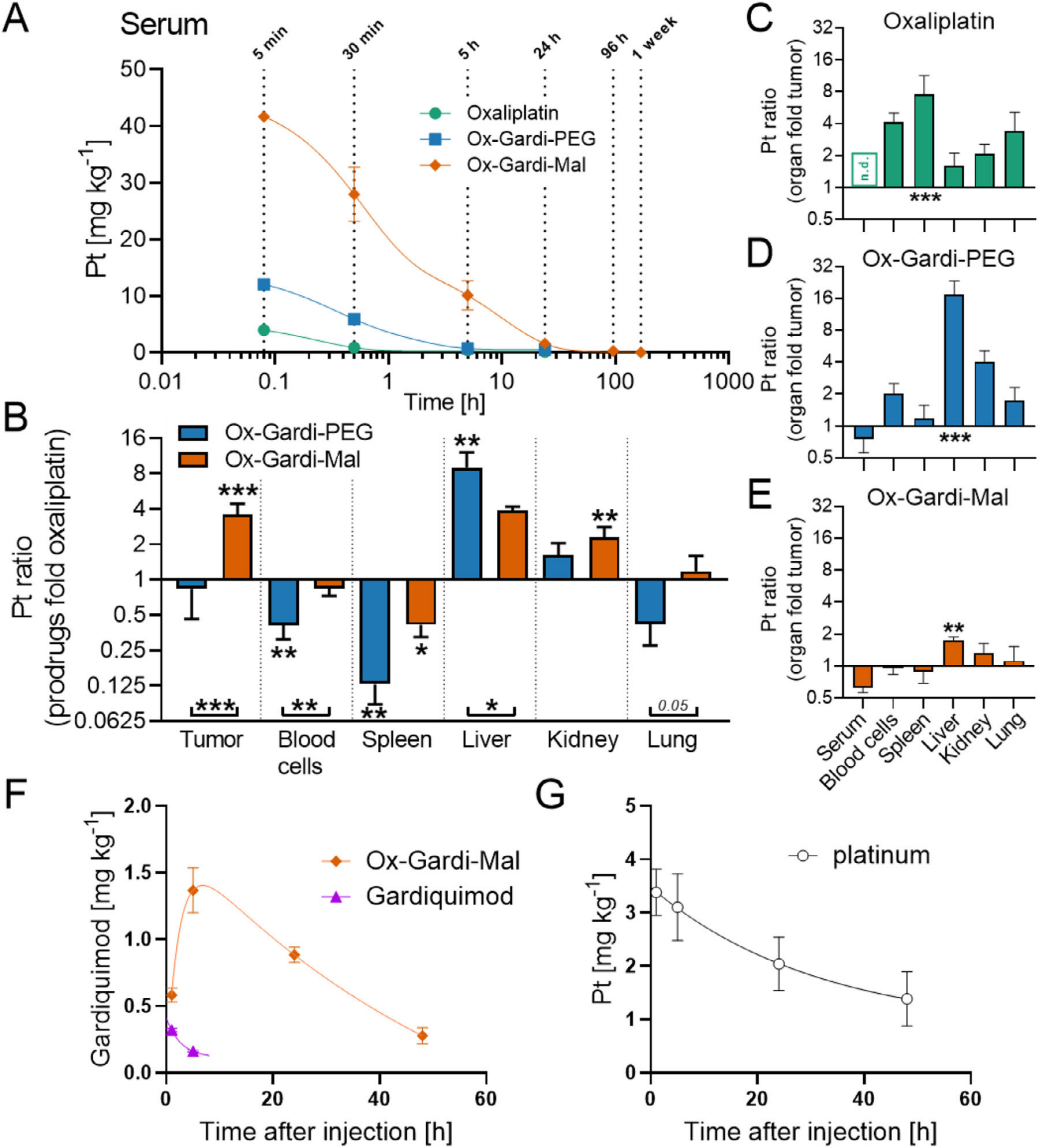
Serum pharmacokinetics and platinum organ distribution of the platinum(IV) prodrugs as compared to oxaliplatin and gardiquimod release in the tumor tissue. (A–E) CT‐26 colorectal cancer‐bearing BALB/c mice (*n* = 4 per group) were dosed i.v. once (A), or twice (B‐E) weekly for 1 week with the indicated drugs, equimolar to 9 mg kg^−1^ oxaliplatin. The underlying treatment regimens are outlined in Figure S14A,G, respectively. Mice were euthanized 7 days after receiving a single dose (A) or 24 h after receiving the second dose (B–E) and platinum contents were measured by ICP‐MS. (A) Platinum levels in serum were determined and analyzed using a two‐phase decay model (compare Figure S14D). (B) Prodrug‐induced platinum levels in the respective organs are given normalized to the levels achieved by oxaliplatin treatment. (C–E) For each indicated compound, platinum levels in the respective organs are depicted normalized to the corresponding tumor tissue. (B–E) The corresponding absolute platinum levels are shown in Figure S14J. Data points are depicted as mean ± SD. The statistical significance of differences in platinum levels between the treatment groups and organs was tested using one‐way ANOVA with the Tukey or Dunnett multiple comparison test. (F,G) CT‐26‐bearing BALB/c mice (*n* = 3 per group) were dosed i.v. once with Ox‐Gardi‐Mal as under (A) or 1 mg kg‐1 gardiquimod followed by analysis of free gardiquimod (F) and total platinum levels for Ox‐Gardi‐Mal‐treated mice (G) at the indicated time points in triplicates by LC‐HRMS and ICP‐MS, respectively. *ICP‐MS* = *Inductively coupled plasma mass spectrometry; i.v. = intravenous; n.d. = not determinable*. *P < 0.05; **P < 0.01; and ***P < 0.001.

Based on the encouraging in vitro results, the in vivo anticancer efficacy of the maleimide‐containing Ox‐Gardi‐Mal was assessed in comparison to the untargeted Ox‐Gardi‐PEG complex and oxaliplatin in a syngeneic colorectal cancer model. For this purpose, BALB/c mice bearing CT‐26 colorectal tumors were dosed via intravenous (i.v.) injection on a twice‐weekly schedule for 2 weeks at doses equimolar to the maximum tolerated dose (MTD) of oxaliplatin (9 mg kg^−1^) (Figure 4A). All investigated platinum‐based drugs significantly reduced tumor burden relative to solvent‐treated mice (calculated up to day 17 from treatment start). Tumor growth inhibition by both prodrugs was superior to oxaliplatin (Figure 4C). The overall survival of mice receiving oxaliplatin was not improved relative to mice receiving solvent, indicated by a median survival of 13 and 14 days, respectively. The non‐maleimide Ox‐Gardi‐PEG prodrug prolonged survival (median: 18.5 days), however, treatment with Ox‐Gardi‐Mal resulted in a more pronounced anticancer effect that translated into significantly prolonged overall survival (median: 25 days) compared to all other compounds tested (Figure 4B). Application of neither oxaliplatin nor Ox‐Gardi‐PEG and Ox‐Gardi‐Mal significantly affected mouse body weight compared to solvent control (Figure S12A). While therapy with the albumin‐targeted prodrug was generally well tolerated, treatment with oxaliplatin as well as the untargeted prodrug was less well manageable, necessitating in some mice premature euthanasia (Figure 4B,C) due to tumor ulceration and, in case of oxaliplatin, also anemia (compare Figure S13C). This was accompanied by a significant reduction in the average splenic weight of oxaliplatin‐treated and, to a lesser extent, Ox‐Gardi‐PEG‐treated mice, but not in mice treated with Ox‐Gardi‐Mal (Figure S12B). Co‐administration of the TLR7/8 inhibitor enpatoran significantly reduced the tumor growth‐inhibiting activity of Ox‐Gardi‐Mal, revealing a pronounced role of TLR7/8 activation in mediating anticancer effects of this platinum(IV) complex (Figure 4D,E; Figure S12C). Considering that the MTD of gardiquimod is around 1 mg kg^−1^ [14, 17, 61], the tolerated i.v. administration of ∼7 mg kg^−1^ gardiquimod in the form of oxaliplatin(IV) complexes underscored the strong potency of albumin‐directed prodrugs in mitigating treatment‐related adverse effects. To elucidate whether the distinct impact of Ox‐Gardi‐Mal on tumor growth and overall survival is based on the prodrug‐mediated constant release of gardiquimod and oxaliplatin in the tumor tissue, we also tested the impact of equimolar application of both free compounds at the MTD of gardiquimod (1 mg kg^−1^) using the identical application scheme as for the platinum(IV) prodrugs (Figure 4F). This dose appeared also meaningful considering the peak concentration of free gardiquimod released in the tumor from Ox‐Gardi‐Mal‐treated mice at around 1.4 mg kg^−1^ tumor tissue (compare Figure 5F). The effects of premixed free gardiquimod and oxaliplatin cotreatment did not reach significance both in terms of tumor growth inhibition and overall animal survival (Figure 4G,H). Interestingly, however, the drug combination showed a clear‐cut trend toward tumor growth inhibition, while both single compounds were completely inactive. This points again toward a synergistic antitumor activity of these two immune‐activating anticancer compounds that can be fully exploited only via a tumor‐specific release of both compounds from a tumor‐targeted prodrug like Ox‐Gardi‐Mal.

### 2.6 Hematological Side Effects

Bone marrow suppression is a well‐recognized side effect of oxaliplatin and commonly associated with anemia.[32] To assess the impact of the prodrugs on hematological parameters, blood from the facial vein of colorectal cancer‐bearing mice was analyzed after receiving a full treatment cycle (Figure S13A) or a single dose of gardiquimod (Figure S13B). Anemia, characterized by a reduction in red blood cells (RBC), hemoglobin (HGB), and hematocrit (HCT), was absent in gardiquimod‐treated animals but particularly pronounced in case of free oxaliplatin, indicating impaired erythropoiesis. A significant decline of platelets (PLT), white blood cells (WBC), granulocytes (GRA) and monocytes (MON) indicated additional oxaliplatin‐mediated myelosuppression in the bone marrow. These hematotoxic effects were in case of all other treatment groups distinctly less pronounced or even absent (Figure S13C). Single‐drug gardiquimod treatment induced pronounced leukopenia, comparable to free oxaliplatin. In Ox‐Gardi‐PEG‐treated mice, lack of any change in immune compartments proved prodrug‐mediated hematoprotection from both free oxaliplatin and gardiquimod. This protection was less complete, but still highly significant, in Ox‐Gardi‐Mal‐treated mice (Figure S13). Considering also that spleen weights remained stable (compare Figure S12B) relative to solvent‐treated mice, the risk of anemia and myelotoxicity appeared to be negligible and manageable in mice receiving therapy with the targeted Ox‐Gardi‐Mal prodrug.

### 2.7 Platinum Organ Distribution and Release of Gardiquimod within the Tumor

A critical parameter in the development of albumin‐targeted prodrugs is the circulatory half‐life, considering that prolonged systemic retention can greatly enhance passive targeting of the TME through the EPR effect and active receptor‐mediated uptake in the tumor tissue. Therefore, we next evaluated the impact of maleimide‐mediated albumin targeting on the tissue distribution of the tumor‐targeted Ox‐Gardi‐Mal prodrug in comparison to the non‐maleimide Ox‐Gardi‐PEG complex and oxaliplatin in CT‐26 colorectal cancer‐bearing mice. The clearance of the compounds from serum and blood cells was determined by measuring platinum content with ICP‐MS. Serum and blood samples were taken from facial vein blood at different time points following application of a single drug dose (Figure S14A). The biodistribution across organs and tumor tissues was monitored after applying a twice‐weekly treatment regimen for 1 week (Figure S14G). In general, application of a single dose, as well as the twice‐weekly drug application was well tolerated and no significant impact on the weight of the mice was observed (Figure S14B,H). While Ox‐Gardi‐Mal therapy significantly reduced tumor burden using both application schemes, Ox‐Gardi‐PEG and oxaliplatin treatment exerted anticancer activity only upon administration of a second dose (Figure S14C,I). Concerning platinum levels in the serum, application of a single dose of Ox‐Gardi‐Mal resulted in a markedly increased AUC with a 9.5‐fold and 29.1‐fold greater total area as compared to treatment with Ox‐Gardi‐PEG and oxaliplatin, respectively (Figure 5A; Figure S14D). Using a two‐phase decay model, the slow‐phase serum half‐life of Ox‐Gardi‐Mal was distinctly extended as compared to Ox‐Gardi‐PEG (6.6‐fold) and oxaliplatin (39.9‐fold). With respect to the fast‐phase half‐life, Ox‐Gardi‐Mal persisted 2.1‐ and 2.5‐fold longer in the serum than Ox‐Gardi‐PEG and oxaliplatin, respectively (Figure S14D). While the highest AUC of platinum levels in circulating blood cells was detected in oxaliplatin‐treated mice (1.8‐ and 1.3‐fold higher AUC compared to Ox‐Gardi‐Mal and Ox‐Gardi‐PEG, respectively), Ox‐Gardi‐Mal showed the fastest blood cell clearance, indicated by distinctly shortened half‐lives (Figure S14E,F).

Next, we assessed the biodistribution of the investigated drugs across organs 24 h after mice had received a second dose within 1 week (Figure 5B–E; Figure S14J). Platinum levels in serum (Figure S14J) and blood cells (Figure 5B; Figure S14J) confirmed the findings from the longitudinal pharmacokinetic study (compare Figure S14A–F), demonstrating enhanced serum retention of the maleimide‐functionalized complex. Concerning the tumor‐targeting strategy, in mice treated with the albumin‐targeted Ox‐Gardi‐Mal complex, significantly higher platinum levels were observed in the tumor tissues compared to oxaliplatin‐ or Ox‐Gardi‐PEG‐treated mice (Figure 5B; Figure S14J). This demonstrates the preferential targeting of the malignant tissue by maleimide‐functionalized complexes as previously reported [41, 42, 43]. Administration of oxaliplatin resulted in the highest platinum accumulation in spleen tissue, compared to the other treatment groups (Figure 5B; Figure S14J). When comparing the organ‐to‐tumor ratio (Figure 5C–E), hyperaccumulation of oxaliplatin‐derived platinum in the spleen became especially apparent (Figure 5C). This finding aligns with the previously observed spleen atrophy in the anticancer efficacy study (compare Figure S12B). Treatment with Ox‐Gardi‐PEG led to reduced splenic accumulation compared to oxaliplatin, which corresponded with less pronounced splenic degradation (compare Figure S12B), as well as lack of anemia (compare Figure S13C). Although splenic platinum load was significantly higher following application of Ox‐Gardi‐Mal compared to Ox‐Gardi‐PEG (Figure 5B), spleen weights in mice receiving the targeted prodrug remained stable (Figure S12B), highlighting the advantage of albumin‐targeting in mitigating toxic side effects. In the liver and kidney tissues of mice receiving Ox‐Gardi‐PEG and Ox‐Gardi‐Mal, respectively, platinum levels were significantly elevated compared to those in oxaliplatin‐treated mice (Figure 5B; Figure S14J). However, in case of the albumin‐targeted prodrug hepatic platinum accumulation was distinctly less pronounced than with the untargeted compound (Figure 5D,E). Nevertheless, we monitored liver and kidney function by determining the serum levels of alanine transaminase (ALT) and aspartate transaminase (AST), as well as blood urea nitrogen (BUN) and creatinine, respectively (Figure S15A,B). While AST and BUN remained unaltered between the treatment groups and creatinine was undetectable, treatment with Ox‐Gardi‐Mal moderately but significantly elevated ALT levels compared to solvent‐ and Ox‐Gardi‐PEG‐, but not oxaliplatin‐treated animals. However, these values were still in the published reference range for ALT levels in BALB/c mice (Figure S15B) [62]. Accordingly, histological assessment revealed no significant alterations in the liver, kidney, spleen, and lung tissues of treated mice (Figure S15C). Possible impacts on liver physiology should be checked carefully during the further preclinical evaluation of Ox‐Gardi‐Mal.

To determine the level of Ox‐Gardi‐Mal reduction and ligand release in the malignant tissue, we followed the levels of free gardiquimod as compared to platinum levels in CT26 tumors after a single intravenous dose of the albumin‐targeting platinum(IV) compound from 1 to 48 h (Figure 5F,G). For comparison, a single gardiquimod dose at the MTD of 1 mg kg^−1^ was also analysed. Free gardiquimod led to the highest tumor levels 1 h post application (0.32 mg kg^−1^) followed by a distinct decline already at 5 h. In contrast, Ox‐Gardi‐Mal‐derived gardiquimod levels in the tumor increased in this time frame to a peak concentration of about 1.4 mg kg^−1^ after 5 h followed by a slow decline with 0.28 mg kg^−1^ gardiquimod still detectable 48 h after application. The respective AUC for Ox‐Gardi‐Mal was ∼40‐fold higher than that of free gardiquimod although the platinum(IV) compound contained only a 7.4‐fold increased molar amount of gardiquimod compared to the 1 mg kg^−1^ dose. The platinum content in the tumor was highest 1 h after Ox‐Gardi‐Mal application (3.4 mg kg^−1^) and declined slowly and constantly with still around 40% of the peak platinum content detectable after 48 h (Figure 5G). Together, these findings established a markedly prolonged half‐life, along with enhanced tumor‐specific accumulation of the maleimide‐containing complex, and prove a distinctly enhanced tumor tissue exposure to platinum and released gardiquimod. This correlates with a significantly improved overall survival and an ameliorated hematological side effect profile compared to the untargeted prodrug and particularly oxaliplatin.

### 2.8 Immune Activation

**FIGURE 6:**
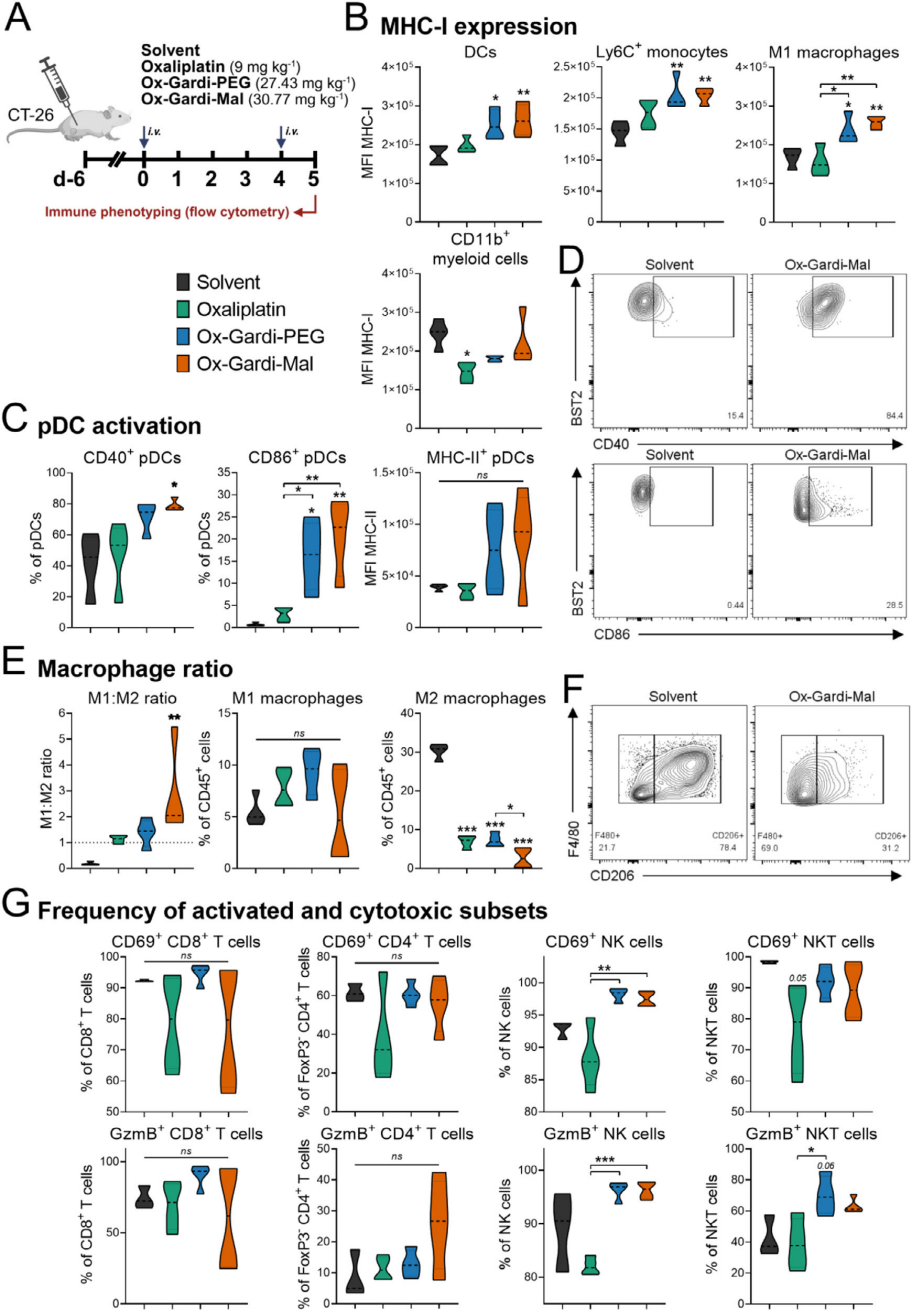
Composition and activation of immune cells in CT‐26 tumors treated with Ox‐Gardi‐PEG and Ox‐Gardi‐Mal, compared to oxaliplatin, analyzed by spectral flow cytometry. (A) CT‐26 colorectal cancer‐bearing BALB/c mice (*n* = 4 per group) were dosed i.v. twice weekly for 1 week with the indicated drugs, equimolar to 9 mg kg^−1^ oxaliplatin. Mice were euthanized 24 h after the second dose. Experimental scheme was created with BioRender.com. (B) Median fluorescence intensity (MFI) of MHC‐I on tumor‐infiltrating immune cells. (C) Frequency of plasmacytoid dendritic cells (pDCs) expressing the activation markers CD40, CD86, and MHC‐II. (D) Representative flow cytometry plots showing the frequency of CD40^+^ and CD86^+^ pDCs. (E) M1:M2 macrophage ratio and frequency of M1 (F4/80^+^ CD206^−^) and M2 (F4/80^+^ CD206^+^) macrophages. (F) Representative flow cytometry plots showing the frequency of M1 and M2 tumor‐associated macrophages. (G) Frequency of activated (CD69^+^) and cytotoxic (granzyme B^+^) immune cell subsets. Data are depicted as violin plots (the median and quartiles are shown by bold and dotted lines, respectively). The statistical significance of differences in frequencies and expression levels between the indicated treatment groups was tested using one‐way ANOVA with the Tukey multiple comparison test. *i.v. = intravenous; MFI = Median fluorescence intensity; DCs = dendritic cells; pDCs = plasmacytoid dendritic cells; NK(T) = Natural killer (T) cells; GzmB = granzyme B*. *P < 0.05; **P < 0.01; and ***P < 0.001.

Next, we evaluated the composition and activation state of immune cells in the tumor and spleen of CT‐26‐bearing mice, following a twice‐weekly treatment regimen for 1 week (Figure 6A). The gating strategies used to identify the respective immune cell subsets and markers are outlined in Figures S16 and S17.

Overall, the frequency of tumor‐infiltrating immune cells or subsets did not differ significantly between the treatment groups, except for an increased CD8^+^ T cell population that reached significance upon treatment with the albumin‐targeted Ox‐Gardi‐Mal prodrug (Figure S18A). This is in line with the known ability of both, oxaliplatin [63] and TLR7/8 agonists [11, 64], to promote CD8^+^ T cell infiltration. In parallel, upregulation of MHC‐I expression on monocytes, macrophages, and DCs was observed in both prodrug‐treated groups, but not in oxaliplatin‐treated mice, which even showed reduced MHC‐I expression on myeloid cells (Figure 6B). This suggests an enhanced fidelity of tumor antigen cross‐presentation via MHC‐I in the prodrug‐treated mice [65]. Although the intratumoral frequency of pDCs remained unchanged in Ox‐Gardi‐PEG‐ and Ox‐Gardi‐Mal‐treated mice (Figure S18A), these cells were highly activated only in the prodrug‐treated animals, as shown by an increased CD40 and CD86 as well as MHC‐II expression (Figure 6C,D). This suggests enhanced maturation and activation of pDCs [66], particularly upon treatment with Ox‐Gardi‐Mal. Comparable observations have been published for topical application of TLR7/8 agonists [11]. In accordance with a more pro‐inflammatory TME, the M1(F4/80^+^, CD206^−^):M2(F4/80^+^, CD206^+^) tumor‐associated macrophage (TAM) ratio was significantly elevated, based on efficient downregulation of M2 in Ox‐Gardi‐Mal‐treated mice, but not in the other treatment groups (Figure 6E,F). A shift in the macrophage polarization toward the tumoricidal M1 subtype was also reported concerning TLR7/8 agonists in combination with platinum‐based chemotherapy [19, 25, 27, 39]. Tumor‐infiltrating CD8^+^ and CD4^+^ T cells were highly activated (CD69 expression) and cytotoxic (granzyme B expression) across all experimental groups, but NK and NKT cells were less activated in oxaliplatin‐treated compared to prodrug‐treated tumors (Figure 6G). In addition, tumor immune cell infiltration was spatially assessed by multiplex immunofluorescence analysis, which confirmed enhanced CD8^+^ T cell abundance in Ox‐Gardi‐Mal‐treated tumors and elevated infiltration of GzmB^+^ cells by both prodrugs and oxaliplatin (Figure S19). Inhomogeneous accumulation of CD8+ T cells under Ox‐Gardi‐Mal treatment might indicate the beginning formation of TLS as observed under combination of cisplatin and the TLR7/8/9 agonist CR108 [13]. Selective downregulation of M2‐like TAMs (F4/80^+^, CD206^+^) in tumor tissues of animals treated with Ox‐Gardi‐Mal (Figure S20) supported the flow cytometry findings (compare Figure 6; Figure S18).

In the spleen, pronounced immunomodulatory differences were observed between the treatment groups (Figures S18B and S21). Treatment with oxaliplatin increased the frequency of splenic CD45^+^ immune cells, in particular CD8^+^ and CD4^+^ T cells, while the abundance of monocytes and DCs was reduced as compared to the other examined platinum(IV) compounds (Figure S18B). Interestingly, mice treated with the Ox‐Gardi‐PEG prodrug exhibited significantly lowered CD45^+^ immune cells in the spleen, compared to the other therapies. Both, Ox‐Gardi‐PEG‐ and Ox‐Gardi‐Mal treatment, induced upregulation of MHC‐I among splenic CD45^+^ cells, especially in B cells and myeloid cells, including monocytes, macrophages, DCs, and pDCs, unlike oxaliplatin or solvent treatments (Figure S21A). Similar to the observation in the tumor tissue, the frequency of activated CD40^+^ and CD86^+^ pDCs with high MHC‐II expression further indicated strong activation of this cell type in the spleen of prodrug‐treated mice (Figure S21B). B cells in mice exposed to Ox‐Gardi‐PEG or Ox‐Gardi‐Mal exhibited marked increase in CD69 and CD86 expression (Figure S21C,D), while the overall B cell frequency remained unchanged between all groups (Figure S21B). Splenic NK cell frequency was reduced in all drug treatment groups compared to solvent, whereas NKT cell abundance observed for the prodrug‐treated groups was exclusively lowered in oxaliplatin‐treated mice (Figure S18B). However, NK and NKT cells, as well as CD8^+^ and CD4^+^ T cells in Ox‐Gardi‐PEG‐ and Ox‐Gardi‐Mal‐treated mice exhibited markedly heightened markers of activation (CD69) and cytotoxicity (granzyme B), compared to oxaliplatin or solvent treatments (Figure S21E,F). Ox‐Gardi‐Mal treatment was associated with moderately elevated regulatory T cells (T_regs_) among CD45^+^ cells in the spleen but not tumor tissue (Figure S18A,B), which could be a peripheral compensatory response to immune activation. Notably, the frequency of T cell immunoglobulin and mucin‐domain‐containing‐3 (TIM‐3)‐expressing, immunosuppressive myeloid cells in the spleen was significantly lower in both, the Ox‐Gardi‐PEG and Ox‐Gardi‐Mal group, compared to the oxaliplatin or solvent application (Figure S21G,H). TIM‐3^+^ myeloid cells correlate with immunosuppression and poor clinical outcomes in colorectal cancer [67, 68], and TLR7/8 agonists reduce TIM‐3^+^ myeloid cells by activating NF‐κB and IRF signaling, thereby driving M2‐to‐M1 macrophage polarization and overcoming oxaliplatin resistance in colorectal cancer [27, 69].

Taken together, these findings are well in agreement with the effects of combined TLR7/8 agonist and platinum‐based therapy that lead to systemic immune cell activation (CD40, CD86, MHC‐II), a shift toward a pro‐inflammatory M1 macrophage phenotype, and enhanced activation and cytotoxicity of CD8^+^ and CD4^+^ T cells, as well as NK and NKT cells.[19, 22, 24, 26, 27] Hence, the delivery of a potent TLR7/8 agonist via albumin‐targeted oxaliplatin(IV) prodrugs represents a promising strategy to modulate the TME toward TLR7/8‐driven inflammation while mitigating toxicities associated with systemic administration of free oxaliplatin and gardiquimod.

### 2.9 Peripheral and Intratumoral Cytokine Secretion

Subsequently, we measured cytokine and chemokine secretion by a multiplex cytokine assay using the experimental regimen from the immune phenotyping experiment (Figure S22A,B). In general, the analysis revealed substantial differences between the prodrugs and oxaliplatin or gardiquimod as single agent (Figure S22). Treatment with gardiquimod significantly increased peripheral secretion of interferon‐gamma (IFN‐γ), interleukin‐2 (IL‐2), IL‐12/IL‐23p40, IL‐15, chemokine (C‐X‐C motif) ligand 10 (CXCL10), as well as macrophage colony‐stimulating factor (M‐CSF) as compared to therapy with the other investigated drugs. Secretion of CCL5 and CXCL11 was induced in mice treated with both, Ox‐Gardi‐PEG and Ox‐Gardi‐Mal, but not in the other treatment groups. The anti‐inflammatory IL‐4 and the closely related IL‐13 were produced predominantly following application of Ox‐Gardi‐PEG and Ox‐Gardi‐Mal, respectively, but were not secreted in gardiquimod‐ or oxaliplatin‐treated mice (Figure S22C,D). Within the tumor tissues, IFN‐γ was significantly induced in animals treated with the Ox‐Gardi‐Mal prodrug and remained non‐significantly altered by Ox‐Gardi‐PEG and oxaliplatin (Figure S22E). While intratumoral levels of IL‐12 were not modulated by the investigated drugs, secretion of IL‐2 and TNF‐α were highest in mice treated with the Ox‐Gardi‐Mal complex, although not reaching significance compared to the other groups (Figure S22E). Attenuated T_H_1 responses in the periphery and tumor‐localized proinflammatory conditions induced by Ox‐Gardi‐PEG and Ox‐Gardi‐Mal substantiates the advantage of the prodrug system in mitigating systemic TLR7/8‐driven cytokine release and immune hyperactivation.

## 3 Conclusion

Harnessing activation of innate immune responses, e.g., via TLR7/8 agonists, holds significant potential as an anticancer agent alone or in combination with chemotherapeutics, including platinum‐based compounds. TLR7/8 agonists are known to induce potent and durable antitumor immunity, however, the clinical application of the only approved TLR7 agonist, imiquimod, is limited to topical use due to the occurrence of severe immune‐related toxicities at systemic application.

In this study, we have developed an albumin‐targeted oxaliplatin(IV) prodrug as a tumor‐specific delivery and activation system for improved immunochemotherapy, combining the TLR7/8 agonist gardiquimod and the ICD inducer oxaliplatin. Albumin‐binding mediated via a maleimide moiety in the second axial position enabled systemic application of the complex and provided a mechanism for controlled TLR7/8 agonist release. Strikingly, our prodrug strategy enabled systemic application of gardiquimod doses by far exceeding the MTD of the free TLR7/8 agonist [14, 17, 61]. Generally, binding of the complex to albumin increases the circulatory half‐life, enables enhanced passive targeting of the TME through the EPR effect, and promotes active uptake into the tumor tissue via albumin receptors on tumor cells or the glycoprotein 60 (GP60) receptor on vascular endothelial cells [35, 36, 70, 71]. Prodrug application resulted in significantly prolonged overall survival in a syngeneic mouse model of colorectal cancer and was accompanied by a hyperaccumulation of the platinum complex and prolonged gardiquimod release within the tumor tissue. Treated mice also experienced reduced hematological toxicities, such as bone marrow suppression and anemia, which are frequently associated with oxaliplatin‐based treatment regimens [32]. Organ toxicities remained widely absent, despite the prolonged plasma half‐life of the platinum(IV) complexes. Although liver histology remained unchanged, the minor upregulation of ALT in Ox‐Gardi‐Mal‐ as compared to solvent‐, but not oxaliplatin‐treated animals calls for caution in the further preclinical development. Immune phenotyping and multiplex immunofluorescence imaging demonstrated markedly elevated immune cell activation in the TME of mice treated with the albumin‐targeted prodrug, while excessive systemic cytokine responses typically associated with TLR7/8 activation were absent. In the tumor tissue of mice receiving Ox‐Gardi‐Mal, highly activated pDCs and elevated MHC‐I levels on antigen‐presenting immune cell subsets were observed together with increased tumor‐infiltrating CD8^+^ T cells, likely associated with enhanced antigen (cross) presentation on APCs. Preferential depletion of CD206^+^ M2 TAMs by Ox‐Gardi‐Mal caused an elevated anti‐tumorigenic M1 to M2 macrophage ratio in the tumor tissue, further demonstrating the potential of this compound class to reshape the TME.

Taken together, our prodrug strategy demonstrates that the systemic exposure to a potent TLR7/8 agonist, released from albumin‐targeted oxaliplatin(IV) prodrugs in a spatiotemporally controlled manner, holds significant potential for a new generation of targeted platinum‐based immune‐prodrugs.

## 4 Experimental Section/Methods

### 4.1 Chemical Synthesis and Characterization

**Reagents and Materials**. Potassium tetrachloridoplatinate was purchased from Johnson Matthey (Switzerland) and gardiquimod was purchased from Santa Cruz Biotechnology (Texas). Reactions were conducted under atmospheric conditions and at room temperature (RT) unless stated otherwise. For analytical HPLC measurements and preparative HPLC purification, Milli‐Q water (18.2 MΩ·cm, Merck Milli‐Q Advantage, Darmstadt, Germany) was used. Chemicals and solvents were purchased from commercial suppliers (Sigma‐Aldrich, Merck, Acros, Fluka, and Thermo Fisher Scientific). Oxaliplatin [48], Ox‐(OH_2_) [49], PMal‐PEG‐NH_2_ [44], Ox‐OH‐PEG [44] and Ox‐OH‐PMal [44] were synthesized as described in the literature. Electrospray ionization (ESI) mass spectra were recorded on a Bruker amazon SL ion trap mass spectrometer in the positive and/or negative mode by direct infusion at the Mass Spectrometry Centre of the University of Vienna. One‐ and two‐dimensional ^1^H and DEPT ^13^C spectra were recorded on a Bruker AV Neo 500 or AV III 600 spectrometer in DMSO‐d6. At room temperature at least two conformational isomers were present, thus the spectra were recorded at 80°C. The solvent residual peak was taken as an internal reference. The ^1^H, DEPT ^13^C NMR spectra and UHPLC chromatograms of the final compounds are depicted in Figures S1−S3. Purification by preparative reverse phase (RP) HPLC was performed on an Agilent 1200 series system using a Waters XBridge C18 column (19×250mm). Elemental analysis measurements were carried out on a PerkinElmer 2400 CHN elemental analyzer at the Microanalytical Laboratory of the University of Vienna and are within ± 0.4%, confirming >95% purity. The content of trifluoracetic acid (TFA) and water can vary between different batches of the same compound.

(OC‐6‐44)‐[(1*R*,2*R*)‐1,2‐Cyclohexanediamino]‐[1‐(4‐amino‐2‐((ethylamino)methyl)‐1*H*‐imidazo[4,5‐c]quinolin‐1‐yl)‐2‐methylpropan‐2‐olato]‐oxalato‐[3,6,9,12‐tetraoxatridecan‐1‐amino]platinum(IV) (Ox‐Gardi‐PEG). Ox‐OH‐PEG4 (50 mg, 0.075 mmol) and bis(2,5‐dioxopyrrolidin‐1‐yl) (29 mg, 0.113 mmol, 1.5 eq.) were dissolved in dry DMF (1 mL) under argon at RT and stirred for 3 h in the dark. Gardiquimod (31 mg, 0.098 mmol, 1.3 eq.) was added and the reaction mixture was stirred for another 48 h. DMF was removed under high vacuum (40°C), the crude was purified via preparative RP HPLC (40% MeOH (+0.1% CF_3_COOH) in H_2_O (+0.1% CF_3_COOH); isocratic) and lyophilized to afford the title compound as a white solid (63.3 mg, 84%). ^1^H NMR (500 MHz, DMSO‐d_6_) δ 13.72‐13.42 (bs, 1H, O*H*), 9.63‐9.21 (m, 2H, N*H*_2_), 8.76‐8.21 (m, 5H, N*H*_2_ + C*H*_Ar_), 7.83‐7.77 (m, 1H, C*H*_Ar_), 7.72‐7.64 (m, 1H, C*H*_Ar_), 7.56‐7.49 (m, 1H, C*H*_Ar_), 6.88‐6.89 + 6.33 (bs, 1H, N*H*_Ca_), 5.45‐4.24 (m, 4H, C*H*_2_C═N_Ar_ + N*H*_2,Ar_), 3.52‐3.32 (m, 18H, C*H_2,_*_PEG_ + C *H*_2_CH_3_ + C *H*_2_COH), 3.23 (s, 3H, OC*H*_3_), 3.12‐2.95 (m, 2H, C*H*_2_NH_Ca_), 2.65‐2.46 (m, 2H, C*H*_cHex_), 2.20‐2.06 (m, 2H, C*H*_cHex_), 1.56‐0.86 (m, 13H, C*H*_cHex_ + CH_2_C *H*_3_ + C*H*_3,i‐PrOH_). ^13^C NMR (126 MHz, DMSO‐d_6_) δ 164.0 (*C*ONH_Ca_), 163.5 (*C*O_Ox_), 163.4 (*C*O_Ox_), 163.3 (*C*ON_Ca_), 154.5 (*C*_q,Ar_), 148.8 (*C*_q,Ar_), 136.6 (*C*_q,Ar_), 134.0 (*C*_q,Ar_), 129.4 (*C*H_Ar_), 124.3 (*C*H_Ar_), 123.2 (*C*H_Ar_), 118.5 (*C*H_Ar_), 113.4 (*C*_q,Ar_), 71.2‐69.3 (*C*H_2,PEG_+ *C* H_2_C═N_Ar_ + *C* H_2_COH + *C* H_2_CH_3_), 61.3 (*C*H_cHex_), 60.9 (*C*H_cHex_), 58.0 (O*C*H_3_), 54.7 (CH_2_ *C* OH), 41.4 (*C*H_2,__PEG_), 40.7 (*C*H_2,__PEG_), 40.1‐39.0 (*C*H_2,__PEG_ under DMSO peak), 31.0 (*C*H_cHex_), 27.6 (CH_2_ *C* H_3_), 23.5 (*C*H_cHex_), 13.2 (*C*H_3,i‐PrOH_). MS (m/z): calcd. C_36_H_57_N_8_O_13_Pt (M + H)^+^, 1004.37; found, 1004.45. EA calcd C_37_H_50_N_4_O_17_PtS·2TFA·0.5H_2_O: C, 38.71; H, 4.79; N, 9.03. Found: C, 38.71; H, 5.04; N, 8.95.

(OC‐6‐44)‐[(1*R*,2*R*)‐1,2‐Cyclohexanediamino][((4‐amino‐1‐(2‐hydroxy‐2‐methylpropyl)‐1*H*‐imidazo[4,5‐c]quinolin‐2‐yl)methyl)(ethyl)carbamato][oxalato][(14‐(2,5‐dioxo‐2,5‐dihydro‐1*H*‐pyrrol‐1‐yl)‐3,6,9,12‐tetraoxatetradecyl)carbamato]platinum(IV) (Ox‐Gardi‐Mal). Ox‐OH‐PMal (150 mg, 0.17 mmol) and bis(2,5‐dioxopyrrolidin‐1‐yl) (65 mg, 0.26 mmol, 1.5 eq.) were dissolved in dry DMF (2 mL) under Argon at RT and stirred for 3 h in the dark. Gardiquimod (68 mg, 0.22 mmol, 1.3 eq.) was added and the reaction mixture was stirred for another 18 h at RT. Subsequently, the reaction mixture was stirred for 2 h at 90°C in open air. The mixture was cooled down to 40°C and DMF was removed under high vacuum. The crude was purified via preparative RP HPLC (38% MeOH (+0.1% CF_3_COOH) in H_2_O (+0.1% CF_3_COOH); isocratic) and lyophilized to obtain the title compound as a white solid (101 mg, 54%). ^1^H NMR (500 MHz, DMSO‐d_6_) δ 13.73‐13.43 (bs, 1H, O*H*), 8.71‐8.21 (m, 5H, N*H*_2_ + C*H*_Ar_), 7.83‐7.77 (d, J = 8.2 Hz, 1H, C*H*_Ar_), 7.72‐7.63 (t, J = 7.6 Hz, 1H, C*H*_Ar_), 7.56‐7.48 (t, J = 7.7 Hz, 1H, C*H*_Ar_), 6.96 (s, 2H, C*H*_Mal_), 6.64‐6.17 (bs, 1H, N*H*_Ca_), 5.01‐4.53 (m, 4H, C*H*_2_C═N_Ar_ + N*H*_2,Ar_), 3.58‐3.52 (m, 6H, C*H*_2_N(CO)_2_ + C*H_2,_*_PEG_), 3.48‐3.36 (m, 16H, C*H*_2,PEG_ + C *H*_2_CH_3_ + C *H*_2_COH), 3.13‐3.04 (m, 2H, C*H*_2_NH_Ca_), 2.68‐2.56 (m, 2H, C*H*_cHex_), 2.24‐2.14 (m, 2H, C*H*_cHex_), 1.57‐0.91 (m, 12H, C*H*_cHex_+ CH_2_C *H*_3_ + C*H*_3,i‐PrOH_). ^13^C NMR (126 MHz, DMSO‐d_6_) δ 170.9 (2x*C*O_Mal_), 164.0 (*C*ONH_Ca_), 163.5 (*C*O_Ox_), 163.4 (*C*O_Ox_), 163.3 (*C*ON_Ca_), 148.7 (2x*C*_q,Ar_), 136.6 (*C*_q,Ar_), 134.6 (2x*C*H_Mal_), 133.9 (*C*_q,Ar_), 129.4 (*C*H_Ar_), 124.3 (*C*H_Ar_), 123.2 (*C*H_Ar_), 118.4 (*C*H_Ar_), 113.3 (*C*_q,Ar_), 70.6‐66.9 (*C*H_2,PEG_ + *C* H_2_C═N_Ar_+ *C* H_2_COH + *C* H_2_CH_3_), 61.4 (*C*H_cHex_), 60.9 (*C*H_cHex_), 54.7 (*C*H_cHex_), 41.4 (*C*H_2,PEG_), 40.7 (*C*H_2,PEG_), 40.1‐39.0 (*C*H_2,PEG_), 36.8 (*C*H_2,PEG_), 31.0 (*C*H_cHex_), 27.5 (CH_2_ *C* H_3_), 23.5 (*C*H_cHex_), 13.2 (*C*H_3,i‐PrOH_). MS (m/z): calcd. C_41_H_60_N_9_O_15_Pt (M + H)^+^, 1113.39; found, 1113.50. EA calcd C_41_H_59_N_9_O_15_Pt·2TFA·0.5H_2_O: C, 40.03; H, 4.63; N, 9.34. Found: C, 40.02; H, 4.64; N, 9.19.

### 4.2 Solid Material Stability of Ox‐Gardi‐PEG and Ox‐Gardi‐Mal

Ox‐Gardi‐Mal and Ox‐Gardi‐PEG were aliquoted into Eppendorf tubes and stored at –20°C, 4°C and 40°C. At 0 h, 24 h, 1 and 4 weeks, one aliquot of each was dissolved to a final concentration of 1 mM in 0.1% TFA and analyzed via HPLC. HPLC runs were performed using a Waters Acquity UPLC HSS T3 column (100 Å, 1.8 µm, 3.0 mm × 50 mm) on a Thermo Scientific Dionex UltiMate 3000 UHPLC‐system equipped with a Waters Acquity UPLC HSS T3 column (100 Å, 1.8 µm, 3.0 mm × 50 mm). Milli‐Q water (mobile phase A) and acetonitrile (ACN) (mobile phase B), both containing 0.1% TFA, were used as eluents. The flow rate was consistent at 0.6 mL/min for all measurements using the following a gradient: 0–0.5 min linear gradient from A 99:1 B to A 99:1 B, 0.50–5.50 min linear gradient to A 1:99 B, 5.50–6.50 min A 1:99 B, 6.50–6.50 min linear gradient to A 99:1 B, 6.5–8.00 min A 99:1 B. Chromatograms were evaluated at a wavelength of 220 nm.

### 4.3 Stability and Reduction Experiments by UHPLC

Each platinum compound was dissolved in Milli‐Q water to obtain a stock solution with a concentration of 10 mM. For stability and reduction kinetics, Ox‐Gardi‐Mal was incubated with 1 equivalent of GSH for 5 min to generate the Ox‐Gardi‐Mal‐GS adduct. Both platinum compounds were subsequently diluted with phosphate buffer (PB, 150 mM, pH 7.4) to a final concentration of 1 mM and incubated at 20°C, with and without 10 eq. AA. The reaction was monitored on the same instrument as the solid material stability tests (see above) using a Waters Acquity UPLC HSS T3 column (100 Å, 1.8 µm, 3.0 mm × 50 mm). Milli‐Q water (mobile phase A) and ACN (mobile phase B), both containing 0.1% TFA, were used as eluents. The flow rate was consistent at 0.6 mL/min for all measurements using the following gradient of 1%–99% acetonitrile over 5 min. At each time point of the reaction, the peak area of the parental complex was integrated at a wavelength of 220 nm.

### 4.4 Albumin‐Binding Properties by SEC‐ICP‐MS

FCS was purchased from Sigma‐Aldrich and buffered to pH 7.4 with 150 mM PB to ensure a stable pH. The platinum(IV) complexes were dissolved in 150 mM PB (pH 7.4) and diluted 1:50 in the buffered serum to obtain a final concentration of 50 or 100 µM. The samples were then incubated in the autosampler at 37°C and analyzed at the 0, 1, 4, 23, and 24 h time point. Between each sample a pure water blank was measured. For SEC‐ICP‐MS measurements an Agilent 1260 Infinity system coupled to an Agilent 7800 ICP‐MS equipped with a dynamic reaction cell was used. Oxygen (purity 5.5, Messer Austria GmbH, Gumpoldskirchen, Austria) was used as reaction gas. The following operation parameters were used: Acquity UPLC BEH column (200Å 1.7 µm, 4.6x150 mm) and CH_3_COONH_4_ (50 mM, pH 6.8) as eluent, at a flow rate of 400 µl min^−1^. The column and sample temperature was kept at 37°C and 0.5 µL were injected.

### 4.5 Analysis of Albumin‐Ox‐Gardi‐Mal Conjugate

The sample preparation was performed in analogy to the procedure described above for “albumin‐binding properties by SEC‐ICP‐MS”. Shortly, Ox‐Gardi‐Mal was dissolved in Milli‐Q water and subsequently diluted 1:50 in the buffered FCS to yield a final concentration of 50 µM. The sample was incubated at 37°C and analyzed after 2 h of incubation. As a reference, buffered FCS without Ox‐Gardi‐Mal was used. For SEC measurements the same column, conditions and instrument was used as described above. The albumin containing peak was collected (elution time between 3.50 and 4.00 min) and 50 µL injected onto a Waters BioResolve Reversed‐Phase Monoclonal Antibody Polyphenyl analytical column (2.1 × 150 mm, 2.7 µm, 450Å) using a Vanquish Horizon UHPLC system coupled to an Orbitrap QExactive mass spectrometer (Thermo Fisher Scientific) at the Mass Spectrometry Centre of the University of Vienna. The column oven temperature was set to 40°C. H_2_O (mobile phase A) and ACN/H_2_O 9:1 (mobile phase B), both containing 0.1% formic acid (FA), were used as eluents. The flow rate was consistent at 0.4 mL min‐1 using the following gradient: 0–20.0 min linear gradient from A 85:15 B to A 40:60 B, 20.0–20.3 min linear gradient to A 10:90 B, 20.3–21.3 min A 10:90 B, 21.3–21.6 min linear gradient to A 85:15 B, 21.6–26.0 min A 85:15 B. The high‐resolution MS spectra were recorded in positive ion mode in the range m/z 700–3000 at a resolution of 17500 (FWHM at 200m/z). The following HESI ion source settings were applied: electrospray voltage: 3.5 kV, ion transfer capillary temperature: 275°C. Charge state determination and deconvolution of ESI mass‐to‐charge ratio spectra and determination of the molecular mass of the sample was performed using the MagTran software program [72].

### 4.6 Incubation with Cell and Tumor Lysates

Cell extracts were prepared from A2780 cells (∼ 10^8^ cells), by incubating cell pellets in liquid nitrogen for 5 min. Cell extracts were then thawed on ice, washed with water, centrifuged (300 × g, 5 min, 4°C), and the pellet was resuspended in 100 µL water at 0.5 × 10^6^ cells mL^−1^. Per well, 5 µL of cell extract, derived from 25 × 10^4^ cells, was added. For tumor lysates, CT‐26 tumors were subcutaneously injected (0.5 × 10^6^ cells in 50 µL of serum‐free RPMI‐1640 medium) into the right flank of 12‐week‐old BALB/c mice. At a mean tumor volume of ∼700 mm^3^, tumors were harvested and mechanically dissociated in 1 mL water using the gentleMACS dissociator (Miltenyi Biotec, Gaithersburg, MD, USA). Tumor pieces (around 200 mg) were incubated in liquid nitrogen for 5 min, thawed on ice, centrifuged (300 × g, 5 min, 4°C), and the supernatant was stored at −80°C. Ox‐Gardi‐PEG and Ox‐Gardi‐Mal were dissolved in Milli‐Q water and subsequently diluted 1:10 to yield a final concentration of 50 µM in the lysates. The samples were incubated at 37°C and 20 µL aliquots were taken after 20 h and 48 h. The aliquots were diluted 1:3 using ACN and stored at 4°C for 10 min. Following centrifugation at 4°C with 20 000 × g for 10 min, the supernatant was diluted 1:1 with Milli‐Q water containing 0.1% FA and analyzed via HPLC‐MS. Measurements were performed on an Agilent 1260 Infinity system using a Waters Acquity UPLC HSS T3 Column 50 mm × 3 mm coupled to an Agilent 6230 Time of Flight mass spectrometer. HPLC grade water containing 0.1 % FA, and ACN containing 0.1 % FA were used as eluents. The flow rate was consistent at 0.6 mL min^−1^ using the following gradient: 0–0.50 min linear gradient from A 95:5 B to A 95:5 B, 0.50–5.50 min linear gradient to A 5:95 B, 5.50–7.00 min A 5:95 B, 7.00–7.10 min linear gradient to A 95:5 B, 7.1–9.00 min A 95:5 B. The MS spectra were recorded in positive ion mode in the range m/z 200‐1700 at an acquisition rate of 5 spectra/s. The following ESI ion source settings were applied: electrospray voltage: 3.5 kV, ion transfer capillary temperature: 350°C. To determine the amount of free gardiquimod, the EIC of 314 ± 0.5 m/z was used. Recovery rate: To determine the recovery rate of gardiquimod, the drug was spiked into the lysates and water as a control. After an incubation time of 15 min, gardiquimod was extracted following the description above. The recovery was determined for both lysates at ≥97%. Standard calibration: gardiquimod in Milli‐Q water was spiked into the lysates (final concentrations: 5‐50 µM) and extracted according to the general procedure.

### 4.7 Cell Culture and Reagents

Colorectal cancer cell models (murine CT‐26 (RRID:CVCL_7256) and human HCT116 (RRID:CVCL_0291) and SW480 (RRID_CVCL_0546)) and the human monocytic THP‐1 cells (RRID:CVCL_0006) were purchased from ATCC (Manassas, VA, USA). The human MC‐38 (RRID:CVCL_B288) colorectal cancer cells and A2780 human ovarian cancer cells (CVCL_0135) were purchased from Sigma‐Aldrich. The oxaliplatin‐resistant subline HCT116/OxR was generated as described previously.[53] The human HEK‐Blue^TM^/hTLR7 (RRID:CVCL_IM84) reporter cell line was purchased from Invivogen (San Diego, CA, USA) (#hkb‐htlr7v2). All reagents were obtained from Sigma‐Aldrich (Sigma‐Aldrich, St. Louis, MO, USA), unless stated otherwise. CT‐26 cells were cultured in Dulbecco's Modified Eagle's Medium/Ham's Nutrient Mixture F12 (DMEM/F12, 1:1) (SAFC Biosciences Ltd., UK), MC‐38 cells in DMEM, supplemented with NEAA (0.1 mM) (Thermo Fisher Scientific), sodium pyruvate (1 mM) and L‐glutamine (2 mM), HCT116 and HCT116/OxR cells in McCoy's 5A modified medium (M8403), and SW480 cells in Minimum Essential Medium Eagle (MEM; M0268). HEK‐Blue/hTLR7 cells were cultured in DMEM, supplemented with L‐glutamine (2 mM), Normocin^TM^ (Invivogen), Pen‐Strep (100 U mL^−1^–100 µg mL^−1^). Cells were selected once a week by additionally supplementing Zeocin (Invivogen) and Blasticidin (Invivogen). THP‐1 and A2780 cells were cultured in RPMI‐1640 medium (R6504). All cell culture media were supplemented with 10% FCS (BioWest, Riverside, MO, USA). Cells were incubated under standard conditions (37°C, 5% CO_2_), regularly checked for mycoplasma contamination, and authenticated via short tandem repeat analysis (Microsynth, Vienna, Austria). Oxaliplatin (LCLabs; # O‐7111) was prepared as 10 mM stock in water. Gardiquimod was prepared as 3 mM stock in Milli‐Q water. The TLR7/8 inhibitor enpatoran (M5049) was obtained from MedChemExpress and prepared as 10 mM stock in DMSO. Ox‐Gardi‐PEG and Ox‐Gardi‐Mal were prepared as 100 mM stock in DMF. AA and GSH were prepared freshly in cell culture medium. All drug stocks were stored at −20°C. Dilutions of stocks were prepared in cell culture medium immediately before the experiment. Corresponding dilutions of DMF were used as solvent control.

### 4.8 MTT‐Based Cell Viability Assay

Cell models were seeded (2–2.5 × 10^3^ cells/well) in 96‐well plates. After 24 h recovery, compounds were applied at the indicated concentrations in triplicates. Cell viability was determined following 72 h of continuous drug incubation using an MTT‐based cell viability assay (EZ4U, Biomedica, Vienna, Austria) according to the manufacturer's recommendations. Absorbance was measured at 450 nm (620 nm as reference) using a multimode microplate reader (Tecan Spark, Zurich, Switzerland). Drug responses were normalized relative to solvent‐treated cells. Half‐maximal inhibitory concentrations (IC_50_ values) were derived via interpolation from dose‐response curves, modeled with the four‐parameter logistic (4PL) nonlinear regression model in GraphPad Prism (La Jolla, CA, USA) from at least three independent experiments. Resistance factors for each investigated drug were calculated as IC_50_ ratio between parental and oxaliplatin‐resistant HCT116 cells. Activation was tested by co‐incubating cells with AA (50 µM), GSH (10 mM), or cell extracts (5 µL corresponding to 25 × 10^4^ cells), and was expressed as the IC_50_ ratio between standard and reducing culture conditions. Drug interactions between oxaliplatin and gardiquimod were established using the ZIP synergy scoring model of SynergyFinder (v3.14) [73].

### 4.9 Intracellular Platinum Accumulation by ICP‐MS

Cell models were seeded (1.5–10 × 10^5^ cells/well) in six‐well plates. After 24 h recovery, compounds were applied at the indicated concentrations in triplicates. After 3 h drug exposure, cells were prepared as published previously [43]. In brief, cells were detached, washed twice in HyClone^TM^ Dulbecco's Phosphate‐Buffered Saline (DPBS) (Cytiva, Marlborough, MA, USA), dried at RT overnight, and lysed in 400 µL nitric acid (68% Rotipuran Supra, Carl Roth, Karlsruhe, Germany) for 2 h at RT. The lysate was filled up to 8 mL with Milli‐Q water. The platinum concentration was determined by ICP‐MS analysis. Rhenium served as internal standard for platinum and both standards were derived from CPI International (Amsterdam, The Netherlands). The total platinum content was determined with a quadrupole‐based ICP‐MS instrument Agilent 7800 (Agilent Technologies, Tokyo, Japan) equipped with the Agilent SPS 4 autosampler (Agilent Technologies, Tokyo, Japan) and a MicroMist nebulizer at a sample uptake rate of approximately 0.2 mL min^−1^. A radio frequency power of 1550 W was used as well as nickel cones. Argon was used as plasma gas (15 L min^−1^) and as carrier gas (∼1.1 L min^−1^). The dwell time was set to 0.1 s and the measurements were performed in 12 replicates with 100 sweeps. The Agilent MassHunter software package (Workstation Software, version C.01.04, 2018) was used for data processing. The ratio of the platinum content between oxaliplatin and the platinum(IV) complex (Ox‐Gardi‐PEG or Ox‐Gardi‐Mal) was calculated for each investigated cell model, as well as between parental and oxaliplatin‐resistant HCT116 cells.

### 4.10 HEK‐Blue/hTLR7 Reporter Cell Assay

The HEK‐Blue/hTLR7 reporter cell line contains an inducible secreted embryonic alkaline phosphatase (SEAP) reporter gene for monitoring TLR7‐dependent activation of the NF‐κB/AP‐1. The SEAP reporter is measured in the cell culture supernatant using QUANTI‐Blue^TM^ (Invivogen) solution. Cells were seeded at 5 × 10^4^ cells/well in 96‐well plates in DMEM medium, supplemented with 10% FCS, L‐glutamine and Pen‐Strep. To study TLR7/8 inhibition, cells were seeded in medium additionally supplemented with enpatoran (100 nM). After 24 h recovery, compounds were applied at the indicated concentrations in triplicates. Cell culture supernatant was collected after 18 h drug incubation, and secreted SEAP reporter levels were measured according to the manufacturer's recommendations. In brief, for the detection of secreted SEAP, 20 µL supernatant were transferred into 96‐well plates and 180 µL QUANTI‐Blue^TM^ added per well. Absorbance was measured at 620 nm following 2 h incubation. Half‐maximal effective concentrations (EC_50_ values) were derived via interpolation from dose‐response curves, modeled with the four‐parameter logistic (4PL) nonlinear regression model in GraphPad Prism. Activation, tested in co‐incubation experiments with AA (50 µM) and GSH (10 mM), were determined as AUC ratio between standard and reducing conditions.

### 4.11 In Vivo Syngraft Experiments

Mice were kept in groups of four per cage with sterilized paper‐based environmental enrichment and food and water ad libitum, in a pathogen‐free environment with a controlled 12 h light‐dark cycle. Syngeneic models of colorectal carcinoma were generated via subcutaneous injection of CT‐26 cells (0.5 × 10^6^ cells in 50 µL of serum‐free RPMI‐1640 medium) into the right flank of 12‐week‐old BALB/c mice. Oxaliplatin was dissolved in 5% glucose. Ox‐Gardi‐PEG and Ox‐Gardi‐Mal were dissolved in 0.9% NaCl and diluted in mouse BALB/c serum (33%) (Innovative Grade; Novi, M, USA). All treatment solutions were prepared immediately before i.v. application. Ox‐Gardi‐PEG (27.43 mg kg^−1^) and Ox‐Gardi‐Mal (30.77 mg kg^−1^) were administered equimolar to the maximal tolerated dose of oxaliplatin (= 9 mg kg^−1^). To determine anticancer efficacy of the investigated compounds, treatment of the mice (*n* = 4 – 12 per group) started 4 days after tumor implantation, when all tumors were measurable (>25 mm^3^) using a twice‐weekly treatment scheme for 2 weeks. To assess the contribution of TLR7/8 activation, the TLR7/8 inhibitor enpatoran (M5049, prepared in 10% DMSO/PBS, 1 mg kg^−1^) was co‐applied per os daily for 10 days. Hematological parameters were assessed 72 h following application of the fourth dose. For the longitudinal pharmacokinetic analysis in serum and blood cells, the drug distribution study across organs by ICP‐MS, the organ toxicity (serum parameters and H/E staining of tissue sections), the immune phenotyping experiment by spectral flow cytometry, and spatial immune phenotyping, as well as the cytokine measurement by Luminex, mice (*n* = 4 per group) were treated i.v. 6 days after subcutaneous implantation, when tumors had reached a volume of ∼400mm^3^, with the respective compounds or solvent. Mice received a single dose of the compounds in case of the longitudinal analysis of platinum content in serum, blood cells, and the tumor as well as for the determination of free gardiquimod levels in the tumor tissue. For the analysis of platinum distribution across the organs, organ toxicity, immune phenotyping, and cytokine secretion, mice were treated twice a week for 1 week and euthanized 24 h after receiving the second dose. Tumor size was determined daily via manual caliper measurement. Tumor volume was calculated as tumor volume = (length × diameter × diameter)/2. The overall condition, shape, and activity of the mice was assessed daily. All animal experiments were performed following Federation of European Laboratory Animal Science Associations (FELASA) and ARRIVE guidelines and according to the regulations of the Ethics Committee for the Care and Use of Laboratory Animals at the Medical University Vienna (proposal number BMBWF 2023‐0.122.324). Mice were euthanized via cervical dislocation upon reaching a tumor volume >1500 mm^3^, loss in body weight (>20%), tumor ulceration, or other indications of deteriorated health.

### 4.12 Hematological Assessment

The therapy of tumor‐bearing mice is outlined in the section “In vivo syngraft experiments” described above. In brief, mice received a twice‐weekly treatment scheme for 2 weeks, and 72 h after receiving the last dose blood parameters were analyzed. Tumor‐free mice (*n* = 3 per group) receiving a single dose of gardiquimod i.v. were analyzed in parallel. Peripheral blood was collected from the submandibular vein in K3E K3EDTA tubes (Greiner Bio‐One; Kremsmünster, AUT) on ice and was analyzed for hematological parameters (red blood cells (RBCs), hemoglobin (HGB), hematocrit (HCT), platelets (PLT), white blood cells (WBCs), granulocytes (GRA), monocytes (MO), and lymphocytes (LYM)) using a Vet ABC hematology analyzer (scil Animal Care GmbH, Viernheim, Germany).

### 4.13 Platinum Organ Distribution by ICP‐MS

The therapy of tumor‐bearing mice is outlined in the section “In vivo syngraft experiments” described above. In brief, in case of the longitudinal pharmacokinetic analysis of platinum content in serum and blood cells, mice received a single dose of the investigated compounds and blood was drawn at the time points indicated. For the analysis of platinum across organs, mice were treated twice weekly for 1 week and euthanized 24 h after receiving the second dose. Mouse tissues were processed as described previously [43]. Briefly, serum was isolated from peripheral blood by centrifugation (900 × g, 10 min, 4°C, twice). Tissues were digested in 20% nitric acid and hydrogen peroxide using an open‐vessel graphite digestion system. The platinum concentration was determined by ICP‐MS analysis. For details see “Intracellular platinum accumulation by ICP‐MS”.

### 4.14 Gardiquimod Quantification in Tumor Tissue

To evaluate the release of free gardiquimod from the tumor tissue, CT26 syngraft‐bearing mice (tumor size around 200‐300 mm^3^) were treated i.v. with a single dose of Ox‐Gardi‐Mal (30.77 mg kg^−1^) or gardiquimod (at MTD 1 mg kg^−1^) and after 1 h, 5 h, 24 h, and 48 h (n = 3 per time point) central, non‐necrotic tumor pieces (20–40 mg) were harvested. Tumor pieces were mechanically shred and immediately snap frozen in aqua bidest (1 µl mg^−1^) followed by three freeze thaw cycles separated by 10 min ultrasound sonication (35 kHz) and a final centrifugation at 20.000 × g (4°C, 10 min). The supernatants were used for reversed‐phase high‐performance liquid chromatography–high resolution mass spectrometry (RP‐HPLC–HRMS) sample preparation in analogy to the procedure described above for “Incubation with cell and tumor lysates”. Briefly, three aliquots of each lysate were taken and ACN added at three times the lysate volume, followed by ultrasonication for 3 min and storage at 4°C for 10 min. After centrifugation at 4°C at 20 000 × g for 10 min, the supernatant was diluted 1:1 with Milli‐Q water containing 0.1% FA. Gardiquimod was quantified by RP‐HPLC–HRMS using a C18 column coupled to an Orbitrap mass analyzer. Chromatographic separation was performed on a Horizon Core LC system (Thermo Fisher Scientific) equipped with a C18 analytical column (150 × 2.1 mm, 3 µm particle size; Thermo Fisher Scientific) maintained at 40°C. The mobile phases consisted of (A) water incl. 0.1%vol. formic acid and (B) ACN/water (95+5, v/v) incl. 0.1%vol. FA, delivered at a flow rate of 0.4 mL min^−^^1^ using the following gradient program: 5% B at 0 min, ramped to 40% B over 5 min, ramped to 100% B over 0.5 min, held for 4.5 min, and re‐equilibrated to initial conditions for 5 min. The injection volume was 10 µL. Detection was carried out on an Exploris 120 Orbitrap mass spectrometer (Thermo Fisher Scientific) operated in positive electrospray ionization (ESI+) mode. Gardiquimod was determined and quantified as the protonated molecular ion [M+H]^+^ at m/z 314.1975 with a resolving power of 60,000 (full width at half maximum at m/z 200). Source parameters were set as follows: spray voltage 3.5 kV, sheath gas 40, auxiliary gas 10, sweep gas 1, capillary temperature 275°C, and vaporizer temperature 300°C. Full scan data were acquired over an m/z range of 100‐750 with an AGC target of 106 and maximum injection time of 120 ms. Quantification was performed using extracted ion chromatograms (±3 ppm mass tolerance), and data analysis was conducted using Thermo Excalibur Quan Browser software (version 4.7). Quantification was based on matrix‐matched external calibration in a concentration range of 0.01‐5 µmol gardiquimod L^−^^1^ tumor lysate. All measurements were corrected for the mean of six tumor lysate method blanks and instrumental drift based on repeated analysis of calibration standards every ten samples. Limit of detection (LOD) was 0.003 nmol mL^−^^1^ tumor lysate, determined from six tumor lysate method blanks and calculated as standard deviation × 3.3. Limit of quantitation (LOQ) was 0.01 nmol mL^−^^1^ tumor lysate, determined from six tumor lysate method blanks and calculated as standard deviation × 10. The total platinum content was determined as described above (see “Platinum organ distribution by ICP‐MS”).

### 4.15 Organ Toxicity

The therapy of tumor‐bearing mice is outlined in the section “In vivo syngraft experiments” described above. In brief, mice were treated with the compounds twice (days 0 and 4) and euthanized 24 h after receiving the second dose. Peripheral blood was collected from the submandibular vein in K3E K3EDTA tubes (Greiner Bio‐One; Kremsmünster, AUT) on ice. EDTA‐blood was centrifuged (900 × g, 10 min, 4°C) twice and the supernatant (EDTA‐plasma) was stored at −80°C. Serum parameters of liver (AST, ALT) and kidney (BUN, creatinine) function were determined in EDTA‐plasma by IDEXX BioAnalytics (Columbia, MO, USA). Tissue sections were prepared from formalin‐fixed, paraffin‐embedded organs from treated mice, stained with hematoxylin/eosin (H&E) using standard methods. Slides were scanned (3DHistech ScanII, 20X) and thoroughly analyzed for structural alterations with a focus on signs of inflammation or necrotic events.

### 4.16 Immune Activation by Spectral Flow Cytometry

The therapy of tumor‐bearing mice is outlined in the section “In vivo syngraft experiments” described above. In brief, mice were treated with the compounds twice weekly for 1 week and euthanized 24 h after receiving the second dose. Tissues were processed as described previously [44]. In brief, tumor and spleen tissues were collected and mechanically dissociated in HyClone^TM^ DPBS buffer containing Ca^2+^/Mg^2+^ (Cytiva) and FCS (5%). Tumor samples were digested using collagenase VIII (1 mg mL^−1^) in a HyClone^TM^ DPBS buffer (without Ca^2+^/Mg^2+^, Cytiva) supplemented with DNAse I (1 mg mL^−1^) for 15 min at 37°C under constant shaking. Samples were filtered using 70 µm cell strainers in a DPBS buffer (without Ca^2+^/Mg^2+^) containing FCS (5%), EDTA (5 mM) and DNase I (20 µg mL^−1^). Subsequent washing steps were performed in DPBS buffer (without Ca^2+^/Mg^2+^) containing FCS (5%), EDTA (5 mM) and DNase I (20 µg mL^−1^), unless indicated otherwise. Red blood cells were removed by short incubation with ACK lysis buffer. For blocking, samples were incubated with Ultra‐LEAF purified anti‐mouse CD16/CD32 antibody (clone: 93; article number: #101330; RRID:AB_2783037; dilution: 1:1000) (Biolegend, San Diego, CA, USA) in BD Horizon Brilliant Stain Buffer (#563794; BD Biosciences, San Jose, CA, USA) for 10 min. Next, samples were incubated with an antibody mixture in BD Horizon Brilliant Stain Buffer for 30 min at 4°C. In the staining panel 1, fluorescent dye‐labeled antibodies targeting murine CD3 (17A2; #100203; RRID:AB_312661; 1:100), CD8α (53‐6.7; #100738; RRID:AB_11204079; 1:160), CD19 (6D5; #115528; RRID:AB_493735; 1:800), I‐A/I‐E (MHC‐II; M5/114.15.2; #107620; RRID:AB_493527; 1:800), CD206 (C068C2; #141732; RRID:AB_2565932; 1:160), CD80 (16‐10A1; #104729; RRID:AB_11126141; 1:80), CD86 (GL‐1; #105043; RRID:AB_2566722; 1:320), CD11c (N418; #117312; RRID:AB_389328; 1:400), Ly6C (HK1.4; #128030; RRID:AB_2562617; 1:320), XCR1 (ZET; #148220; RRID:AB_2566410; 1:320), CD317 (BST2; RA3‐6B2; #127010; RRID:AB_1953285; 1:80), CD103 (QA17A24; #156919; RRID:AB_2924488; 1:160), and F4/80 (BM8; #123165; RRID:AB_2894417; 1:80) were purchased from Biolegend. The antibodies targeting murine CD4 (RM4‐5; #85‐0042‐U100; 1:320), CD11b (M1/70; #60‐0112‐U100; RRID:AB_2621836; 1:640), and Ly6G (1A8; #65‐1276‐U100; RRID:AB_2621899; 1:640), as well as the viability dye Ghost Dye Red 780 (#13‐0865‐T100; 1:1000) were purchased from Tonbobio (San Diego, CA, USA). The antibody targeting CD45 (30‐F11; #363‐0451‐82; RRID:AB_2925264; 1:160) was purchased from Thermo Fisher Scientific. The antibodies targeting H‐2Kd (MHC‐I; SF1‐1.1; #749880; RRID:AB_2874120; 1:40), CD40 (3/23; #741749; RRID:AB_2871115; 1:80), and CD172a (SIRPα; P84; #740766; RRID:AB_2740429; 1:160) were purchased from BD Bioscience. In the staining panel 2, fluorescent dye‐labeled antibodies targeting murine CD3 (17A2; #100203; RRID:AB_312661; 1:50), CD8α (53‐6.7; #100738; RRID:AB_11204079; 1:160), CD19 (6D5; #115528; RRID:AB_493735; 1:400), CD49b (DX5; #108924; RRID:AB_2565271; 1:229), CD11b (M1/70; #101228; RRID:AB_893232; 1:80), CD69 (H1.2F3; #104512; RRID:AB_493564; 1:40), I‐A/I‐E (MHC‐II; M5/114.15.2; #107620; RRID:AB_493527; 1:800), and TIM‐3 (RMT3‐23; #119745; RRID:AB_2922462; 1:40) were purchased from Biolegend. The antibody targeting murine CD4 (RM4‐5; # 85‐0042‐U100; 1:80), as well as the viability dye Ghost Dye Red 780 (#13‐0865‐T100; 1:1000) were purchased from Tonbobio. The antibodies targeting CD45 (30‐F11; #363‐0451‐82; RRID:AB_2925264; 1:80) and CD44 (IM7; #365‐0441‐82; AB_2925375; 1:80) were purchased from Thermo Fisher Scientific. Samples incubated with the staining panel 1 were then washed and resuspended in DPBS buffer containing FCS (5%), EDTA (5 mM) and DNase I (20 µg mL^−1^) and then immediately measured on the spectral flow cytometer. Samples incubated with the staining panel 2 were further prepared for intracellular staining of granzyme B (GzmB) (QA16A02; #372208; RRID:AB_2687032; 1:20) and FoxP3 (150D; #320014; RRID:AB_439750; 1:100) using the True‐Nuclear Transcription Factor Buffer Set (Biolegend; #424401) according to the manufacturer's recommendations. In brief, samples were incubated with Perm Fix Solution for 60 min at RT. Next, samples were stained with antibodies targeting GzmB and FoxP3 (diluted in Perm buffer) for 30 min at RT. Then, samples were resuspended in DPBS buffer containing FCS (5%), EDTA (5 mM) and DNase I (20 µg mL^−1^) and incubated overnight at 4°C. The next day, samples were measured on the spectral flow cytometer Cytek Aurora 5L (Cytek Biosciences, CA, USA). Data were analyzed using SpectroFlo (v3.2.1) (Cytek Biosciences) and FlowJo (v10.8.1) (FlowJo LLC, Ashland, OR, USA) software.

### 4.17 Multiplexed Immunofluorescence Analysis

The therapy of tumor‐bearing mice is outlined in the section “In vivo syngraft experiments” above. In brief, CT‐26 xenograft‐bearing mice were treated with the compounds twice (days 0 and 4) and euthanized 24 h after receiving the second dose. Tissue sections were prepared from formalin‐fixed, paraffin‐embedded organs from treated mice using standard methods. Immune cell and activation markers were detected using the Opal 6‐Plex Manual Detection Kit for Whole Slide Imaging (Akoya Biosciences, Marlborough, MA, USA) for multiplex immunofluorescence imaging, according to the manufacturer's recommendations. Antibodies targeting murine CD8 (clone: EPR21769; Abcam, #ab217344; RRID:AB_2890649; 1:500), CD206/MMR (R&D, #AF2535; RRID:AB_ 2063012; 1:100), F4/80 (clone: D2S9R; Cell signaling technology; #70076; RRID:AB_ 2799771; 1:200), and GzmB (clone: 16G6; Thermo Fisher Scientific, #14‐8822‐82; RRID:AB_ 468530; 1:100) were used in the following order and combination: opal 520 1:150 (CD8), opal 570 1:100 (CD206), opal 690 1:100 (F4/80), and opal 620 1:100 (GzmB). Goat anti‐rabbit IgG H&L HRP polymer (#AB214880, Abcam, Cambridge, UK) was used as secondary antibody against CD8 and F4/80. Secondary donkey anti‐goat IgG antibody (Thermo Fisher Scientific; 1:200) was used to detect CD206. Goat anti‐rat IgG H&L HRP polymer (#AB214882, Abcam) was used as secondary antibody against rat GzmB. All steps were performed light‐protected and at RT. In brief, antigens were retrieved in target retrieval solution pH 9 (#S2367, Dako) for 1 h in a steamer at 100°C. Slides were incubated in H_2_O_2_ in PBS for 10 min. Between the subsequent incubation steps, slides were washed with PBS/Tween 0.1% three times. AB diluent/blocking solution (Akoya) was added for 15 min, then the primary antibody was incubated in the same buffer for 45 min at RT. Next, the secondary antibody was incubated for 15 min at RT, then opal dyes (Akoya) were diluted in Amplification diluent (Akoya) and added for 10 min. Next, slides were incubated in antigen retrieval buffer in a steamer for 15 min at 100°C. These steps were repeated for the indicated antibodies. Nuclei were stained with spectral DAPI (Akoya). Prolong Diamond Antifade Mountant (Thermo Fisher Scientific) and cover glasses (No. 1.5H) were used to cover the slides. Slides were scanned (Evident VS200, SpectraSplit 7 filter set) at 20X and analyzed using HALO AI (Indica Labs, Albuquerque, NM, USA) quantitative imaging analysis (v4.2) using the Highplex FL module.

### 4.18 Peripheral and Intratumoral Cytokine Secretion by Luminex

The therapy of tumor‐bearing mice is outlined in the section “In vivo syngraft experiments” described above. In brief, mice were treated with the compounds twice weekly for 1 week and euthanized 24 h after receiving the second dose. Peripheral blood of treated mice was collected from the submandibular vein in K3E K3EDTA‐tubes (Greiner Bio‐One; Kremsmünster, AUT). EDTA‐blood was centrifuged (900 × g, 10 min, 4°C) twice and the supernatant (EDTA‐plasma) was stored at −80°C. Tumor tissues were collected and proteins isolated using a lysis buffer. Tissues were mechanically dissociated using gentleMACS dissociator and were treated with ultrasound for 10 min on ice. Samples were centrifuged (16.800 × g, 15 min, 4°C) and the supernatant was collected. Protein solutions were frozen and the ice sublimated (to dryness) in vacuo. Proteins were then resuspended in universal assay (UAB) buffer from the ProcartaPlex assay (Thermo Fisher Scientific). Quantification of secreted protein targets in plasma and tumor tissues of treated mice was performed using a ProcartaPlex (96‐well) multiplex assay panel (Thermo Fisher Scientific) on the xMAP detection platform (Luminex). The ProcartaPlex (96‐well) multiplex assay panel was performed according to the manufacturer's recommendations, as described previously [44]. In brief, the capture bead mix was incubated with mouse plasma or standard mix overnight. The plasma samples were loaded onto the multiplex assay plate, and biotinylated detection antibody mix and Streptavidin‐PE were added to the plate. Following the recommended washing steps, the samples were incubated in reading buffer, measured on a Luminex xMAP platform (Bio‐Plex MAGPIX Multiplex Reader; Bio‐Rad Laboratories), and analyzed using the xPONENT software.

### 4.19 Data Evaluation and Statistical Analysis

The statistical tests used to determine significance and the corresponding *P* values are described in the respective figure legends. Significance was set at *P* < 0.05. All Student's *t*‐tests are unpaired and two‐sided unless indicated otherwise. One‐way analysis of variance (ANOVA) and two‐way ANOVA was performed with the Tukey multiple comparison test unless indicated otherwise. Statistical tests were conducted using GraphPad Prism version 9.3.0 for Windows (GraphPad Software Inc., San Diego, CA, USA). Error bars represent the standard deviation (SD) unless otherwise indicated.

## Author Contributions

Conceptualization: M.G., M.D., C.R.K., and W.B.; Data curation: M.G., M.D., P.S., A.F., and C.P.; Formal analysis: M.G., M.D., P.S., A.F., and C.J.; Funding acquisition: B.K.K., C.R.K., and W.B.; Investigation: M.G., M.D., P.S., V.K., M.S., P.H., C.R.K., and W.B.; Methodology: M.G., M.D., P.S., J.C., D.B., C.J., I.S., and V.K.; Project administration: M.G., M.D., B.K.K., C.R.K., and W.B.; Resources: J.C., D.B., D.H.B., P.H., C.R.K., and W.B.; Software: M.G., M.D., P.S., A.F., C.J., and C.P.; Supervision: B.K.K., C.R.K., and W.B.; Validation: C.P., M.S., D.H.B., and P.H.; Visualization: M.G., M.D., and I.S.; Roles/Writing – original draft: M.G., M.D., and W.B.; Writing – review & editing: M.G., M.D., P.S., J.C., C.J., C.P., M.S., D.H.B., P.H., C.R.K., and W.B.

## Conflicts of Interest

The authors declare no conflicts of interest.

## Supporting information




**Supporting File**: advs74892‐sup‐0001‐SuppMat.docx.

## Data Availability

The data that support the findings of this study are available from the corresponding authors upon request.
