# Supplementary material for: Improved Systemic Immunochemotherapy Employing an Oxaliplatin‐TLR7/8 Agonist Prodrug Strategy
Source: Adv Sci (Weinh). 2026 Apr 13;13(31):e14588. doi: 10.1002/advs.202514588 (PMC13588035; doi:10.1002/advs.202514588)
Supplement: Supplementary file 1 [file ADVS-13-e14588-s001.docx]

Supporting Information

**Improved Systemic Immunochemotherapy Employing an Oxaliplatin-TLR7/8 Agonist Prodrug Strategy**

*Michael Gutmann^‡^, Martijn Dijkstra^‡^, Philipp Salomon^‡^, Jamie Cowles, Anja Federa, Dina Baier, Carola Jaunecker, Iemima Semerean, Venla Karvonen, Christine Pirker, Maria Sibilia, Dietmar Herndler-Brandstetter, Petra Heffeter, Bernhard K. Keppler, Christian R. Kowol^*^, and Walter Berger^*^*

‡These authors contributed equally to this work; *shared position.

*Email: walter.berger@meduniwien.ac.at

Table of contents

| **Figure** | **Content** |
| --- | --- |
| Figure S1 | ^1^H and ^13^C-NMR spectra of **Ox-Gardi-PEG** |
| Figure S2 | ^1^H and ^13^C-NMR spectra of **Ox-Gardi-Mal** |
| Figure S3 | UHPLC chromatograms of **Ox-Gardi-PEG** and **Ox-Gardi-Mal** |
| Figure S4 | Stability of solid platinum(IV) complexes |
| Figure S5 | Release of gardiquimod and oxaliplatin from Pt(IV) complexes under reducing conditions |
| Figure S6 | Gardiquimod release from platinum(IV) prodrugs |
| Figure S7 | SEC-ICP-MS and HPLC-ESI-MS data |
| Figure S8 | Drug interaction between free oxaliplatin and gardiquimod |
| Figure S9 | Dose-response curves of investigated drugs and impact of reducing agents |
| Figure S10 | Intracellular platinum accumulation in cancer cells by ICP-MS |
| Figure S11 | Impact of TLR7/8 inhibition on gardiquimod-mediated pathway induction by the investigated drugs and impact of reducing conditions |
| Figure S12 | Body and spleen weights of mice treated with the investigated drugs |
| Figure S13 | Hematological parameters |
| Figure S14 | Pharmacokinetics in serum and blood cells, and organ distribution by ICP-MS |
| Figure S15 | Impact of the investigated drugs on liver and kidney serum parameters and histological assessment of organs |
| Figure S16 | Gating strategy for immune cells in tumor tissue |
| Figure S17 | Gating strategy for immune cells in spleen tissue |
| Figure S18 | Frequency of immune cells in tumor and spleen tissue by flow cytometry |
| Figure S19 | Spatial assessment of CD8^+^ and GZMB^+^ immune cells in CT-26 tumors of mice treated with the investigated drugs |
| Figure S20 | Spatial assessment of macrophages in CT-26 tumors of mice treated with the investigated drugs |
| Figure S21 | Systemic immunomodulatory effects of the investigated drugs |
| Figure S22 | Peripheral and intratumoral cytokine secretion in mice treated with the investigated drugs |

**Figure S1.** ^1^H and ^13^C-NMR spectra of **Ox-Gardi-PEG**, measured in DMSO-d6 at 80°C.

**Figure S2.** ^1^H and ^13^C-NMR spectra of **Ox-Gardi-Mal**, measured in DMSO-d6 at 80°C.

**Figure S3**. UHPLC chromatograms of (A) **Ox-Gardi-PEG** and (B) **Ox-Gardi-Mal** at 220 nm.

**Figure S4. Stability of solid platinum(IV) complexes.** Long-term stability studies of solid **Ox-Gardi-PEG** and **Ox-Gardi-Mal** at different time points (day 0, day 1, 1 week and 4 weeks) and temperatures (–20°C, 4°C and 40°C) analyzed by HPLC measurements at wavelength: 220 nm.

**Figure S5. Release of gardiquimod and oxaliplatin from Pt(IV) complexes under reducing conditions and impact of maleimide thiol interaction on maleimide hydrolysis.** HPLC chromatograms of (A) 1 mM Ox-Gardi-PEG in the presence of 10 eq. ascorbic acid (AA), (B) 1 mM Ox-Gardi-Mal in the presence of 10 eq. AA and C) 1 mM Ox-Gardi-Mal first incubated with one equivalent GSH to protect the maleimide moiety and subsequent addition of 10 eq. AA. Each solution was measured immediately after incubation and after 25 h.

**Figure S6. Gardiquimod release from platinum(IV) prodrugs.** (A) Release of gardiquimod from 100 µM Ox-Gardi-PEG or Ox-Gardi-Mal in the presence of 1 mM and 10 mM GSH at 37°C. (B) Release of gardiquimod from 50 µM Ox-Gardi-PEG or Ox-Gardi-Mal in tumor lysates (ex vivo CT-26 samples) and cell lysates (prepared from A2780 cells) after 20 h and 48 h incubation using HPLC-MS.

**Figure S7**. **SEC-ICP-MS and HPLC-ESI-MS measurements**. (A) ^195^Pt-traces of incubation of 100 µM **Ox-Gardi-PEG** in FCS (containing 150 mM phosphate buffer (PB), pH = 7.4) at 37°C over 24 h, measured with SEC-ICP-MS. (B) Sulfur traces of FCS (+150 mM PB, pH 7.4) as well as pure albumin in PB (50 mM, pH 7.4), both measured by SEC-ICP-MS. The small peak at ~3.3 min corresponds to the albumin dimer. (C) HPLC-ESI-MS reference spectrum of the albumin-containing serum fraction and the respective deconvoluted peaks (native albumin at 66427 Da).

**Figure S8. Drug interaction between free oxaliplatin and gardiquimod.** Cell viability was determined by MTT-based cell viability assay. The ZIP synergy scoring model (SynergyFinder) was used to calculate the degree of interaction between oxaliplatin and gardiquimod. One representative experiment out of at least three performed is shown.

**Figure S9. Dose-response curves of Ox-Gardi-PEG and Ox-Gardi-Mal in cancer cell models compared to oxaliplatin and gardiquimod and impact of reducing conditions**. Cell viability was determined by MTT-based cell viability assay. (A-C) Dose-response curves of oxaliplatin, Ox-Gardi-PEG, and Ox-Gardi-Mal under reducing conditions (50 µM AA) and (D, E) gardiquimod ± AA as indicated. (F) Impact of reducing conditions (AA, 50 µM) on the cytotoxicity of gardiquimod, shown as IC_50_ ratio between –AA and +AA. (G-I) Dose-response curves of oxaliplatin, Ox-Gardi-PEG, Ox-Gardi-Mal, and gardiquimod under reducing conditions (10 mM GSH, or cell extracts as indicated). Data points are depicted as mean ± SD. One representative experiment out of at least three performed is shown. The statistical significance of differences in IC_50_ ratios (F, H) was tested using Student’s *t*-test. *IC_50_* *= half-maximal inhibitory concentration; AA = L-ascorbic acid; GSH = glutathione; n.d. = not determinable*. **P* < 0.05 and ***P* < 0.01.

**Figure S10. Intracellular platinum accumulation in cancer cell models treated with Ox-Gardi-PEG or Ox-Gardi-Mal compared to oxaliplatin.** (A-C) Cells were treated with the indicated concentrations of the depicted drugs for 3 h, and cellular platinum contents were measured by ICP-MS. The intracellular platinum content normalized to the cell number is given in (A), the platinum ratio Ox-Gardi-PEG or Ox-Gardi-Mal to oxaliplatin in (B), and the platinum ratio between HCT116/OxR and HCT116 cells in (C). Data points are depicted as mean ± SD. Data are derived from at least three independent experiments in triplicate. The statistical significance of differences in platinum ratios (B) between Ox-Gardi-PEG, Ox-Gardi-Mal, and oxaliplatin was tested using one-way ANOVA with the Tukey multiple comparison test. The statistical significance of differences in platinum ratios (C) between HCT116 and HCT116/OxR cells was tested using Student’s *t*-test. *ICP-MS = Inductively coupled plasma mass spectrometry; Pt = platinum;* *conc. = concentration; LOQ = limit of quantification; n.d. = not determinable*. **P* < 0.05; ***P* < 0.01; and ****P* < 0.001.

**Figure S11. Impact of TLR7/8 inhibition (enpatoran) on gardiquimod-mediated pathway induction by Ox-Gardi-PEG or Ox-Gardi-Mal compared to gardiquimod/oxaliplatin and impact of reducing conditions**. (A-C) NF-κB/AP-1-inducible secreted embryonic alkaline phosphatase (SEAP) reporter levels were determined by measuring the absorbance at 620 nm in the HEK-Blue/hTLR7 cell culture supernatant following 18 h of treatment with the indicated drugs and the TLR7/8 inhibitor enpatoran (100 nM). Cells were incubated with enpatoran for 24 h prior to treatment with the indicated drugs. (A-C) Dose-response curves of the indicated drugs, modelled using the four-parameter logistic (4PL) non-linear regression model, under (A) standard and (B, C) reducing (50 µM AA or 10 mM GSH, as indicated) cell culture conditions. (D) Viability of HEK-Blue/hTLR7 cells treated with the indicated platinum(IV) complexes, at the identical conditions as under (A-C). (E) The impact of enpatoran on HEK-Blue/hTLR7 cell viability was determined at the indicated concentration in a 72 h continuous drug exposure setting by an MTT-based survival assay. (F) HEK-Blue/hTLR7 cell viability for the indicated drugs and their combinations is shown representatively for panel (B). One representative experiment out of at least three performed is shown in (D-F). Data points are depicted as mean ± SD. *AA = L-ascorbic acid; GSH = glutathione*.

**Figure S12 Body and spleen weights of mice treated with Ox-Gardi-PEG or Ox-Gardi-Mal compared to oxaliplatin.** (A) CT-26 colorectal cancer-bearing BALB/c mice were dosed intravenously (i.v.) twice weekly for two weeks with Ox-Gardi-Mal (*n* = 12), Ox-Gardi-PEG (*n* = 4), or oxaliplatin (*n* = 8), equimolar to 9 mg kg^-1^ oxaliplatin, as compared to solvent (*n* = 12). Arrows below the x-axis indicate the days of drug application. Euthanasia of individual mice is indicated by an X symbol in place of a data point symbol. (B) Spleen weights were assessed upon euthanization of the individual mice. (C) CT-26 colorectal cancer-bearing BALB/c mice were dosed intravenously (i.v.) twice weekly for two weeks with Ox-Gardi-Mal (*n* = 4) equimolar to 9 mg kg^-1^ oxaliplatin as compared to solvent (n=4). Mice were treated daily for 10 days with the TLR7/8 inhibitor enpatoran or solvent. The statistical significance of differences in body weights (A, C) (until day 17 from treatment start as indicated by the dashed line) between the treatment groups was tested using two-way ANOVA with the Sidak multiple comparison test. The statistical significance of differences in spleen weights (B) between the indicated drugs was tested using one-way ANOVA with the Tukey multiple comparison test. **P* < 0.05; and ****P* < 0.001.

**Figure S13. Impact of Ox-Gardi-PEG and Ox-Gardi-Mal on hematological parameters compared to oxaliplatin and gardiquimod.** (A, B) CT-26 colorectal cancer-bearing BALB/c mice were dosed i.v. twice weekly for two weeks with Ox-Gardi-Mal (*n* = 12), Ox-Gardi-PEG (*n* = 4), or oxaliplatin (*n* = 8), equimolar to 9 mg kg^-1^ oxaliplatin, compared to solvent (*n* = 12). Gardiquimod (*n* = 3 mice per group) was applied as single dose. Blood was drawn from the submandibular vein at the indicated time point. Experimental schemes were created with BioRender.com. (C) Hematological parameters (RBC, red blood cells; HGB, hemoglobin; HCT, hematocrit; PLT, platelets; WBC, white blood cells; GRA, granulocytes; MON, monocytes; LYM, lymphocytes) were assessed using a Vet ABC hematology analyzer. Data are depicted as violin plots, with the median and quartiles shown by bold and dotted lines, respectively. The statistical significance of differences was tested using one-way ANOVA with the Tukey multiple comparison test. **P* < 0.05; ***P* < 0.01; and ****P* < 0.001.

**Figure S14. Serum and blood cell pharmacokinetics, and platinum organ distribution of Ox-Gardi-PEG or Ox-Gardi-Mal compared to oxaliplatin**. (A-J) CT-26 colorectal cancer-bearing BALB/c mice (*n* = 4 per group) were dosed i.v. once (A-F), or twice (G-J) with the indicated drugs, equimolar to 9 mg kg^-1^ oxaliplatin. Mice were euthanized 7 days after receiving a single dose (A) or 24 h after receiving the second dose (G), and cellular platinum was measured by ICP-MS. Experimental schemes in (A, G) were created with BioRender.com. (B, H) Body weights of the mice are shown relative to treatment start. (C, I) Tumor volumes, assessed via caliper measurements of mice treated with the indicated compounds, are shown as mean ± SEM. (D-F) The AUC of platinum levels and the serum half-life (in h) were calculated by a two-phase non-linear regression model in serum (D) and blood cells (E, F). (J) Absolute platinum levels in the indicated organs are given for each investigated compound. Data points are depicted as mean ± SD. The statistical significance of differences in body weights between the treatment groups was tested using one-way ANOVA with the Tukey multiple comparison test. The statistical significance of differences in tumor volumes between the treatment groups was tested using two-way ANOVA with the Sidak multiple comparison test. *ICP-MS = Inductively coupled plasma mass spectrometry; Pt = platinum; i.v. = intravenous; CI = confidence interval; LOQ = limit of quantification; ns= non-significant*. ***P* < 0.01; and ****P* < 0.001.

**Figure S15. Impact of Ox-Gardi-PEG, Ox-Gardi-Mal, and oxaliplatin on liver and kidney serum parameters and histological assessment of organs.** (A-C) CT-26 colorectal cancer-bearing BALB/c mice were dosed i.v. twice (day 0 and 4) with Ox-Gardi-Mal (*n* = 12), Ox-Gardi-PEG (*n* = 4), or oxaliplatin (*n* = 8), equimolar to 9 mg kg^-1^ oxaliplatin, or solvent (*n* = 12). Tissues were collected 24 h after receiving the second dose. (A) Experimental scheme was created with BioRender.com. (B) Serum parameters of liver (ALT and AST) and kidney function (BUN and creatinine) were determined in EDTA-plasma. Data are depicted as mean ± SEM. (C) Histological sections of the indicated tissues stained with H&E are depicted. The statistical significance of differences was tested using one-way ANOVA with the Tukey multiple comparison test. ***P* < 0.01. *ALT = alanine transaminase; AST = aspartate transaminase; BUN = blood urea nitrogen; LOQ = limit of quantification; ns = non-significant.*

**Figure S16**. **Gating strategy for the identification of CD45^+^ immune cells and immune cell subsets in CT-26 tumors.** (A-B) Representative flow cytometry plots showing the gating strategy for identifying CD45^+^ immune cells and immune cell subsets using panel 1 (A) and panel 2 (B). *RBCs = red blood cells; FoxP3 = forkhead box P3; NK(T) = Natural killer (T) cells; DCs = dendritic cells; pDCs = plasmacytoid dendritic cells*.

**Figure S17. Gating strategy for the identification of CD45^+^ immune cells and immune cell subsets in the spleen.** (A-B) Representative flow cytometry plots showing the gating strategy for identifying CD45^+^ immune cells and immune cell subsets using panel 1 (A) and panel 2 (B). *RBCs = red blood cells; FoxP3 = forkhead box P3; NK(T) = Natural killer (T) cells; DCs = dendritic cells; pDCs = plasmacytoid dendritic cells*.

**Figure S18. Frequency of immune cells in CT-26 tumors and spleen tissues of mice treated with Ox-Gardi-PEG or Ox-Gardi-Mal compared to oxaliplatin**. (A-B) CT-26 colorectal cancer-bearing BALB/c mice (*n* = 4 per group) were dosed i.v. twice (day 0 and 4) with the indicated drugs, equimolar to 9 mg kg^-1^ oxaliplatin. The underlying treatment regimen is outlined in Figure 6A. Mice were euthanized 24 h after the second dose and tumor, as well as spleen tissues were analyzed by spectral flow cytometry. Frequency of CD45^+^ immune cells, as well as innate and adaptive immune cell subsets in CT-26 tumors (A) and spleen (B) are shown. Data are depicted as violin plots, with the median and quartiles shown by bold and dotted lines, respectively. *Treg = regulatory T cells; NK(T) = Natural killer (T) cells; DCs = dendritic cells; pDCs = plasmacytoid dendritic cells*. **P* < 0.05; ***P* < 0.01; and ****P* < 0.001.

**Figure S19. Spatial assessment of CD8^+^ and GZMB^+^ immune cells in CT-26 tumors of mice treated with Ox-Gardi-PEG or Ox-Gardi-Mal as compared to oxaliplatin**. (A-B) CT-26 colorectal cancer-bearing BALB/c mice (*n* = 4 per group) were dosed i.v. twice (day 0 and 4) with the indicated drugs, equimolar to 9 mg kg^-1^ oxaliplatin. The underlying treatment regimen is outlined in Figure 6A. Mice were euthanized 24 h after the second dose and multiplex immunofluorescence analysis was performed on tumor sections. (A) Photomicrographs of representative regions within the tumor tissue. (B) Frequency of CD8^+^, GZMB^+^, CD8^+^/GZMB^+^, and CD8^-^/GZMB^+^ immune cells in CT-26 tumors evaluated by HALO imaging software are shown. Data are depicted as mean ± SEM. The statistical significance of differences was tested using Student’s *t*-test. *GZMB = granzyme B*. **P* < 0.05; ***P* < 0.01; and ****P* < 0.001.

**Figure S20. Spatial assessment of macrophages in CT-26 tumors of mice treated with Ox-Gardi-PEG or Ox-Gardi-Mal compared to oxaliplatin**. (A-B) CT-26 colorectal cancer-bearing BALB/c mice (*n* = 4 per group) were dosed i.v. twice (day 0 and 4) with the indicated drugs, equimolar to 9 mg kg^-1^ oxaliplatin. The underlying treatment regimen is outlined in Figure 6A. Mice were euthanized 24 h after the second dose and multiplex immunofluorescence analysis was performed on tumor sections. (A) Photomicrographs of representative regions within the tumor tissue. (B) Frequency of F4/80^+^, F4/80^+^/CD206^+^, and F4/80^+^/CD206^-^ macrophages in CT-26 tumors evaluated by HALO imaging software are shown. Data are depicted as mean ± SEM. The statistical significance of differences was tested using Student’s *t*-test. ***P* < 0.01.

**Figure S21. Systemic immunomodulatory effects of Ox-Gardi-PEG and Ox-Gardi-Mal compared to oxaliplatin.** (A-B) CT-26 colorectal cancer-bearing BALB/c mice (*n* = 4 per group) were dosed i.v. (day 0 and 4) with the indicated drugs, equimolar to 9 mg kg^-1^ oxaliplatin. The underlying treatment regimen is outlined in Figure 6A. Mice were euthanized 24 h after the second dose and spleen tissues were analyzed by spectral flow cytometry. (A) Median fluorescence intensity (MFI) of MHC-I on immune cells. (B) Frequency of plasmacytoid dendritic cells (pDCs) expressing the activation markers CD40, CD86 and MHC-II. (C) Frequency of B cells expressing the activation markers CD69 and CD86. (D) Representative flow cytometry plots showing the frequency of CD69^+^ and CD86^+^ B cells. (E) Frequency of activated (CD69) and cytotoxic (granzyme B) immune cell subsets. (F) Representative flow cytometry plots showing CD69 and granzyme B expression in CD8^+^ T cells. (G) Frequency of TIM-3^+^ myeloid cells are shown among the myeloid immune cell subset. (H) MFI of TIM-3 on myeloid cells. Data are depicted as violin plots (the median and quartiles are shown by bold and dotted lines, respectively). The statistical significance of differences in frequencies and expression levels between the indicated treatment groups was tested using one-way ANOVA with the Tukey multiple comparison test. *i.v. = intravenous; MFI = Median fluorescence intensity; DCs = dendritic cells; pDCs = plasmacytoid dendritic cells; NK(T) = Natural killer (T) cells; GzmB = granzyme B*. **P* < 0.05; ***P* < 0.01; and ****P* < 0.001.

**Figure S22. Peripheral and intratumoral cytokine secretion in mice treated with Ox-Gardi-PEG or Ox-Gardi-Mal as compared to oxaliplatin and gardiquimod**. Cytokines were detected using a Luminex multiplex platform. (A, B) CT-26 colorectal cancer-bearing BALB/c mice (*n* = 4 per group) were dosed i.v. (day 0 and 4) with the indicated drugs, equimolar to 9 mg kg^-1^ oxaliplatin. Gardiquimod (*n* = 3) was applied as single dose. Experimental schemes were created with BioRender.com. (C, D) Blood was drawn 24 h after the last dose from the submandibular vein. Plasma was isolated and analyzed for secreted cytokines and chemokines. (C) Absolute levels of the indicated cytokines in the plasma are given. (D) Cytokine levels for the respective treatments are shown normalized to the solvent-treated mice, with significant values underlined. (E) Absolute amounts of the indicated cytokines in the tumor tissues are shown. Data points are depicted as mean ± SD. The statistical significance of differences in cytokine levels between the treatment groups was tested using one-way ANOVA with the Tukey multiple comparison test (C, D) or Student’s *t*-test (E). *IFN- γ = Interferon-gamma; IL = Interleukin; CCL5 = CC-chemokine ligand 5; CXCL = chemokine (C-X-C motif) ligand; M-CSF = macrophage colony-stimulating factor; TNF-α = tumor necrosis factor*. **P* < 0.05; ***P* < 0.01; and ****P* < 0.001.
